# Supplementary figures and images for: Homeostatic control of an iron repressor in a GI tract resident
Source: eLife. 2023 May 25;12:e86075. doi: 10.7554/eLife.86075 (PMC10259491; doi:10.7554/eLife.86075)

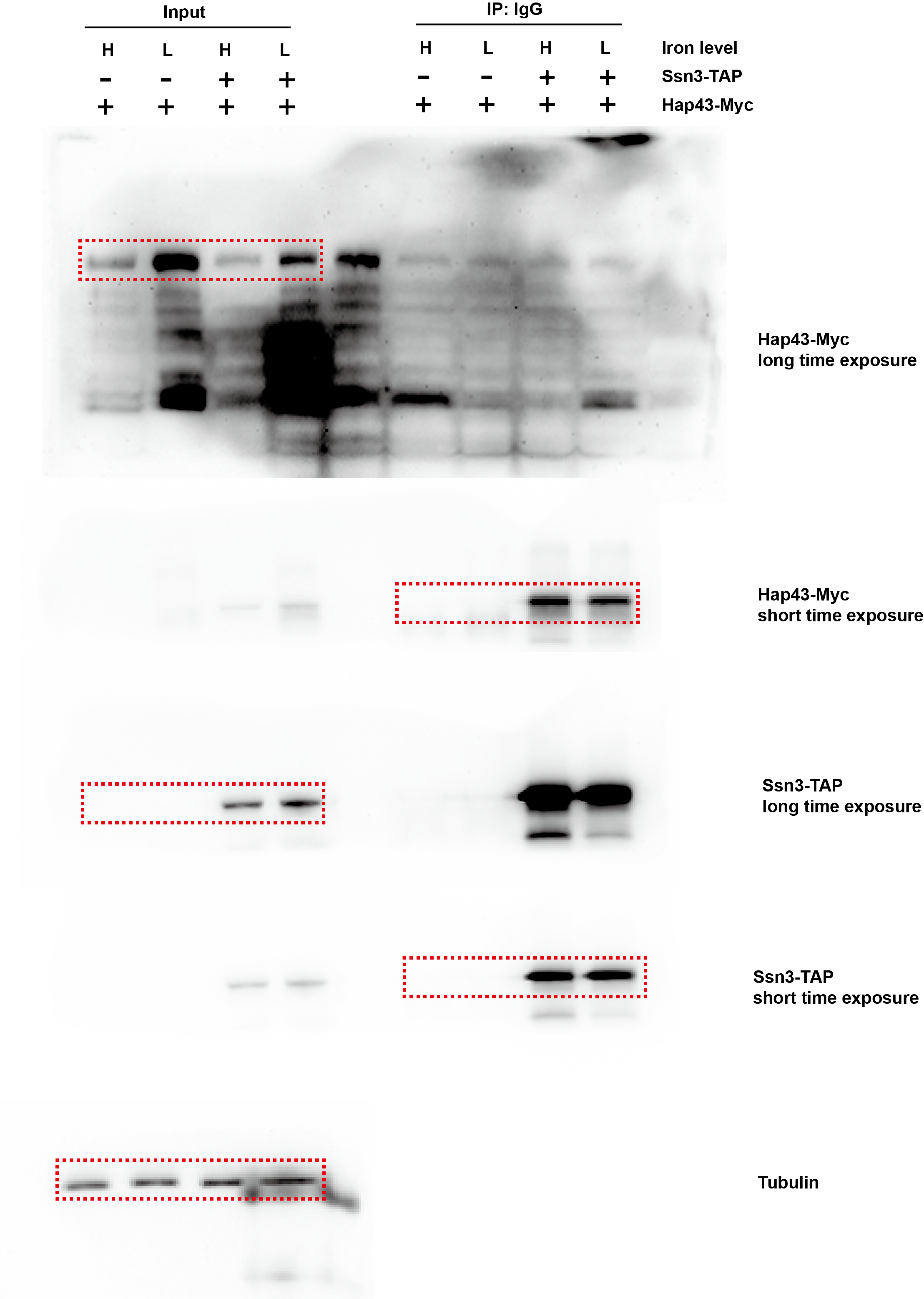

Supplement: Figure 2—source data 1. [file elife-86075-fig2-data1.zip › Figure 2-source data/F/Figure 2F with the uncropped gels or blots.tif]

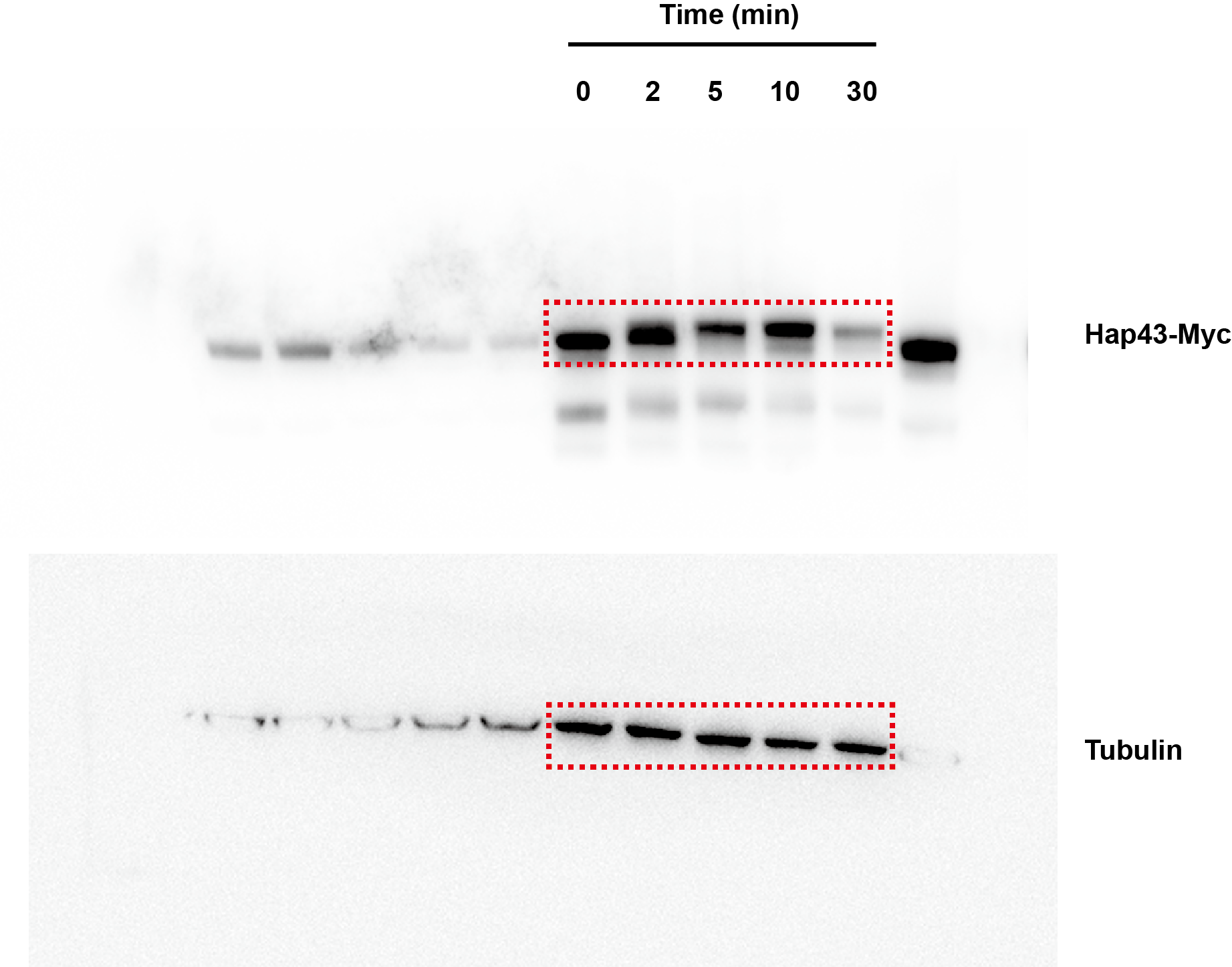

Supplement: Figure 2—source data 1. [file elife-86075-fig2-data1.zip › Figure 2-source data/C/Figure 2C with uncropped gels or blots.tif]

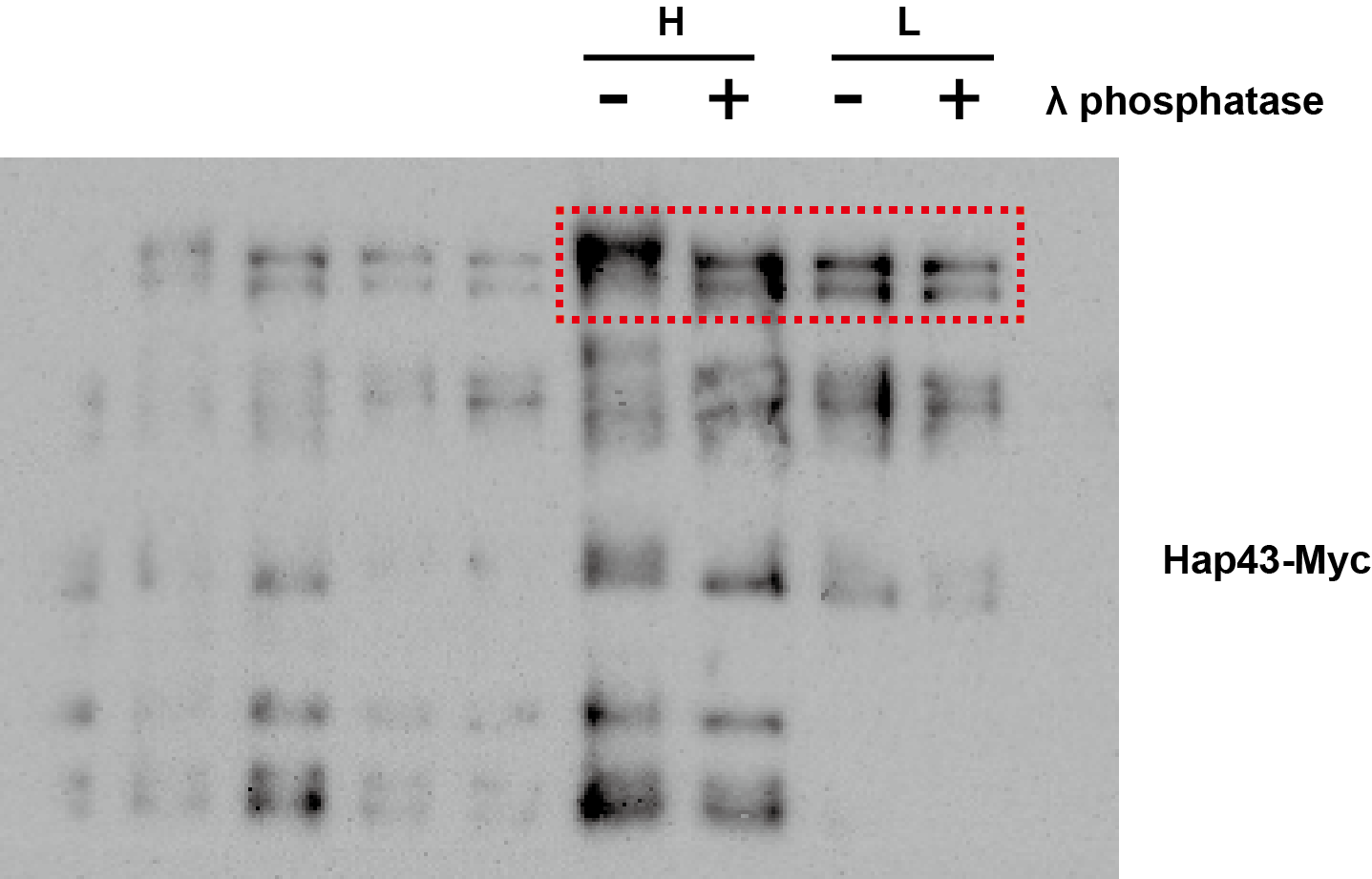

Supplement: Figure 2—source data 1. [file elife-86075-fig2-data1.zip › Figure 2-source data/D/Figure 2D with the uncropped gels or blots.tif]

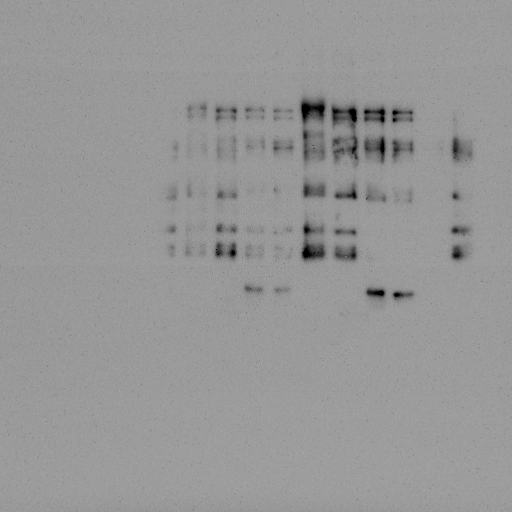

Supplement: Figure 2—source data 1. [file elife-86075-fig2-data1.zip › Figure 2-source data/D/raw unedited gels or blots.tif]

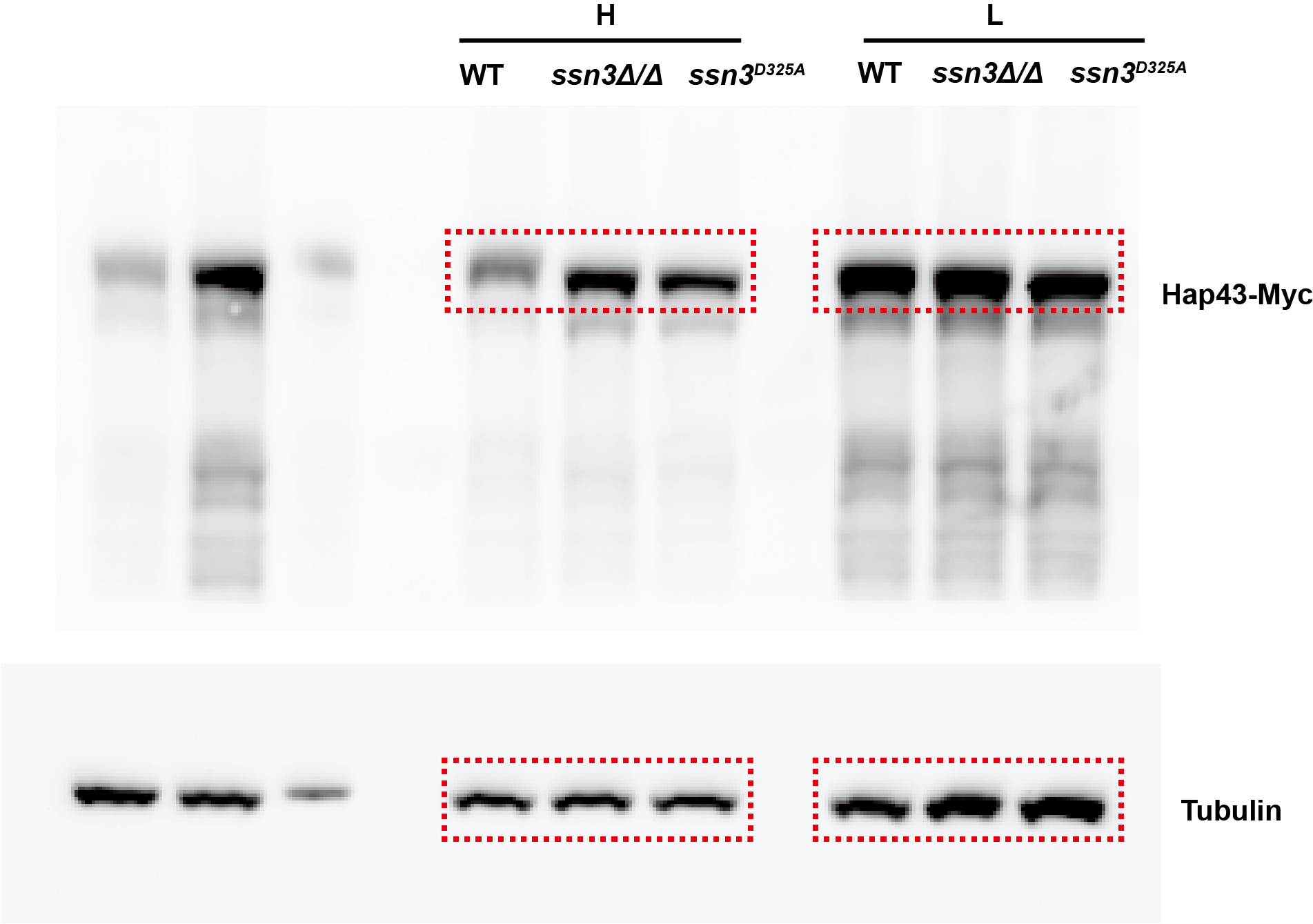

Supplement: Figure 2—source data 1. [file elife-86075-fig2-data1.zip › Figure 2-source data/E/Figure 2E with the uncropped gels or blots.tif]

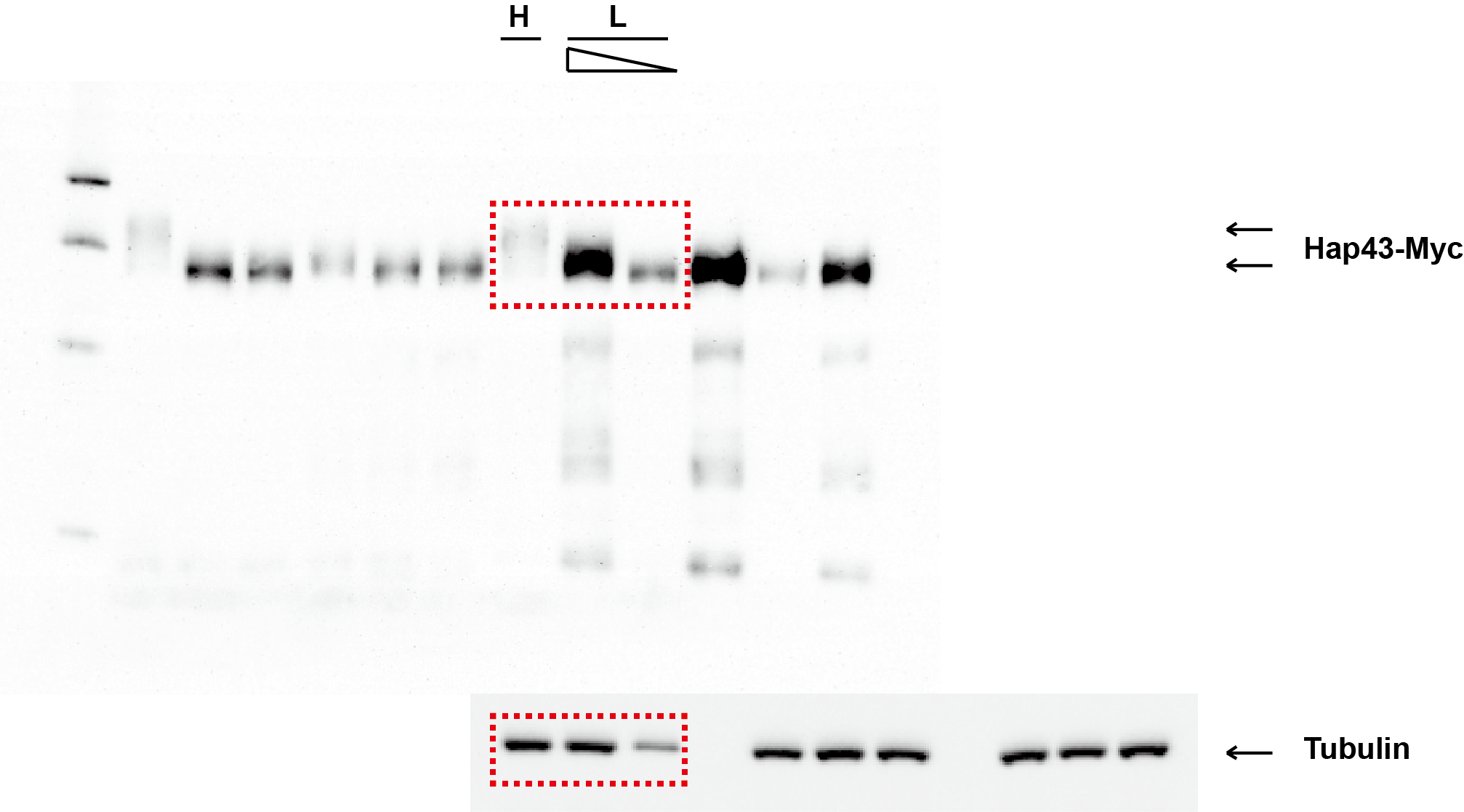

Supplement: Figure 2—source data 1. [file elife-86075-fig2-data1.zip › Figure 2-source data/B/Figure 2B with uncropped gels or blots.tif]

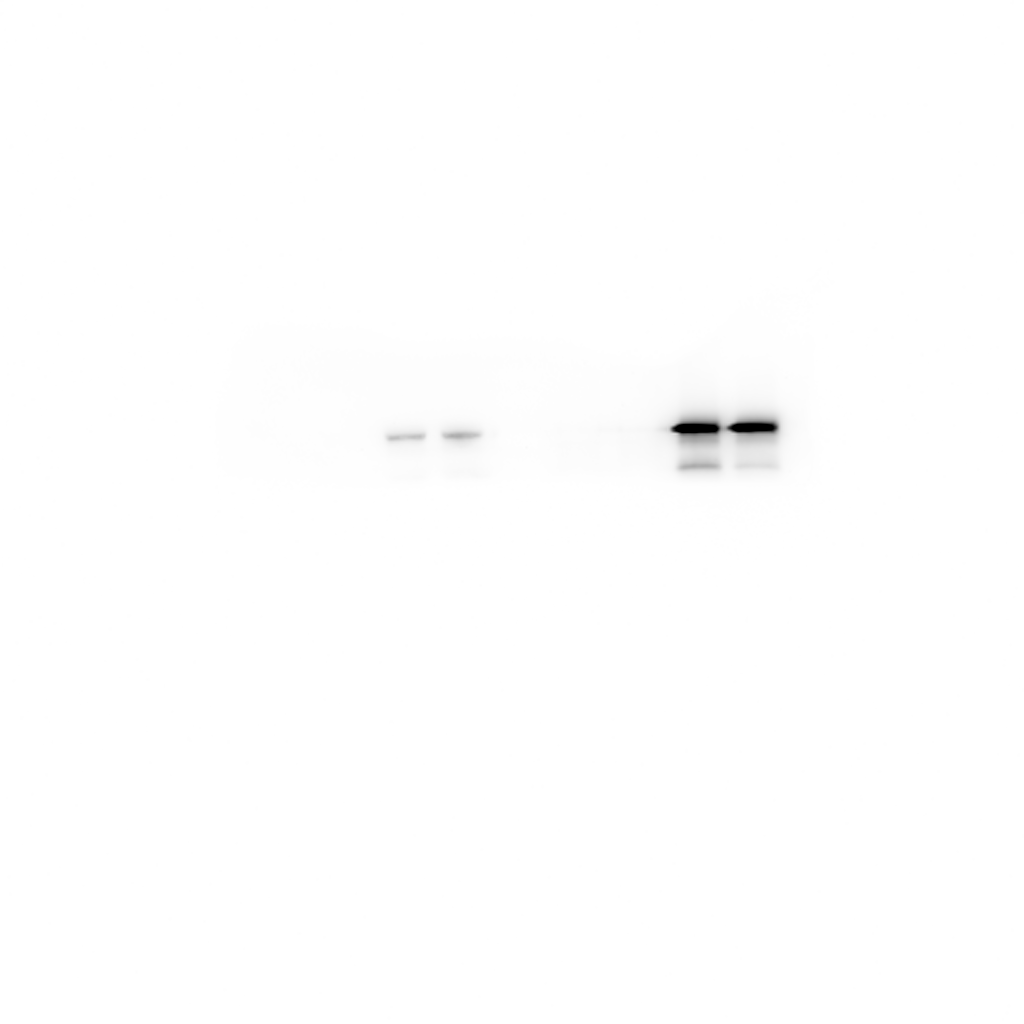

Supplement: Figure 2—source data 1. [file elife-86075-fig2-data1.zip › Figure 2-source data/F/raw unedited gels or blots/TAP with short time exposure.tif]

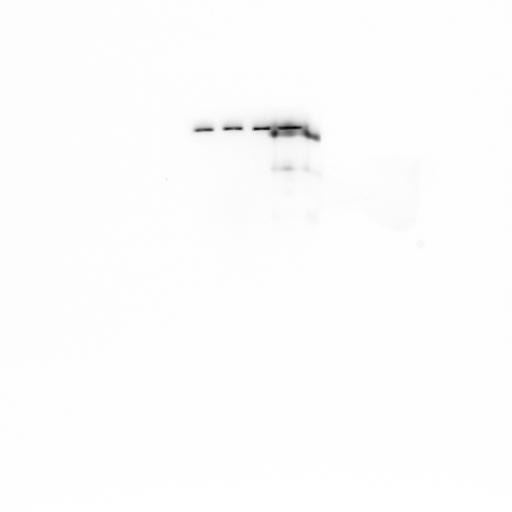

Supplement: Figure 2—source data 1. [file elife-86075-fig2-data1.zip › Figure 2-source data/F/raw unedited gels or blots/Tubulin.tif]

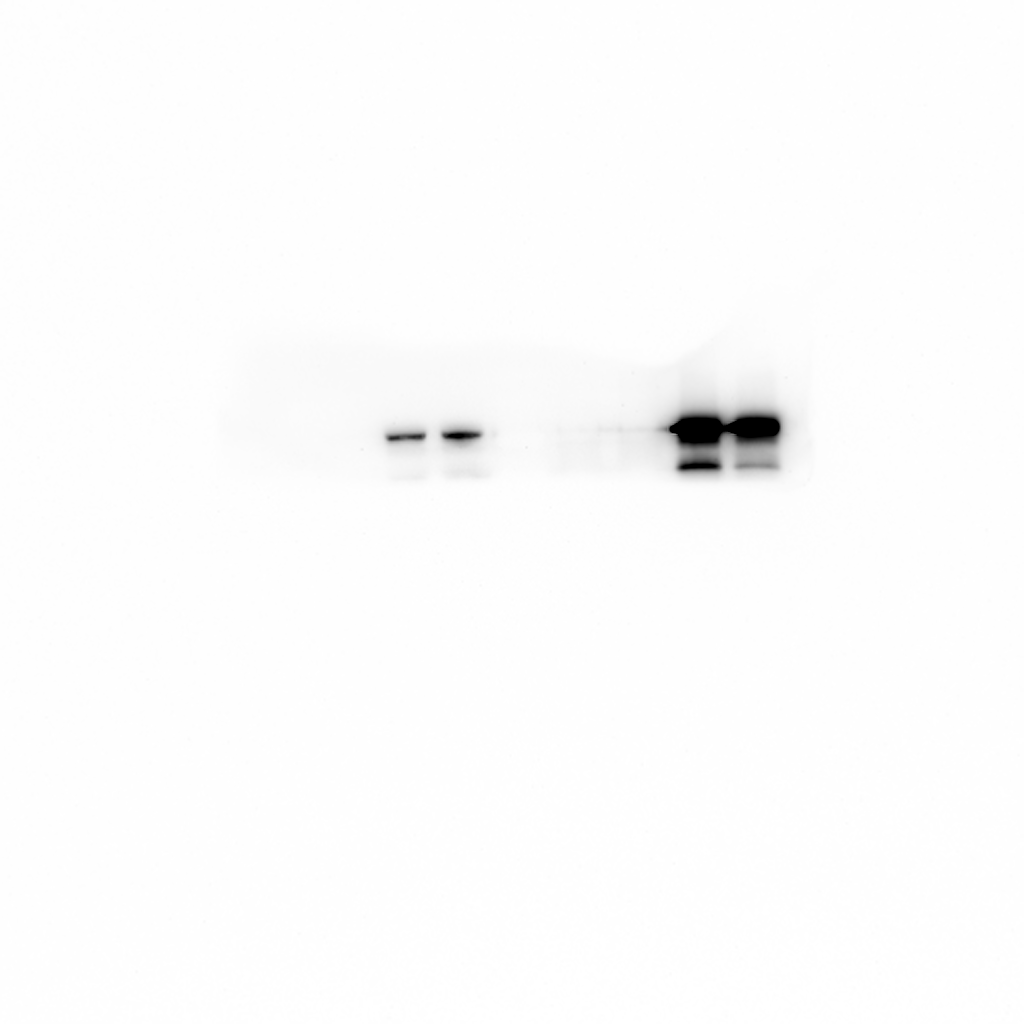

Supplement: Figure 2—source data 1. [file elife-86075-fig2-data1.zip › Figure 2-source data/F/raw unedited gels or blots/TAP with long time exposure.tif]

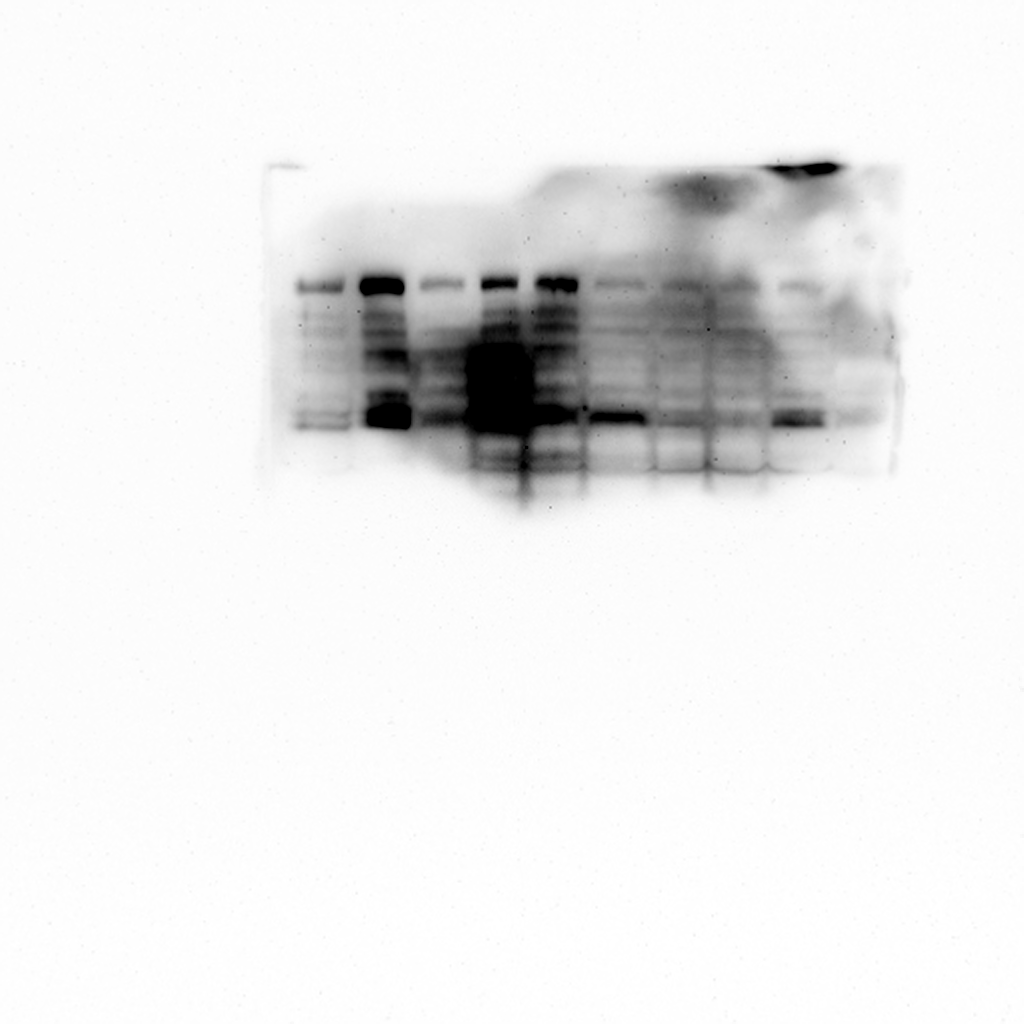

Supplement: Figure 2—source data 1. [file elife-86075-fig2-data1.zip › Figure 2-source data/F/raw unedited gels or blots/Myc with long time exposure.tif]

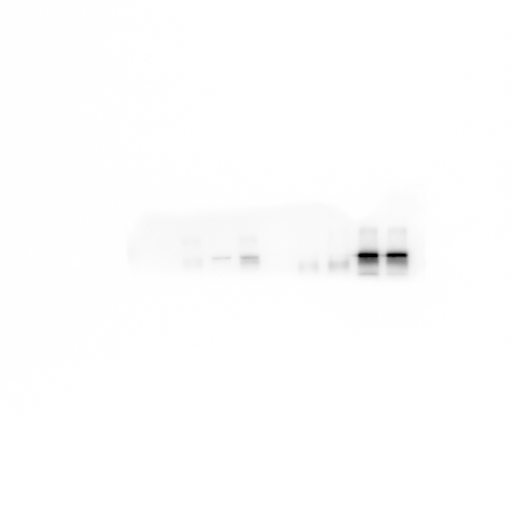

Supplement: Figure 2—source data 1. [file elife-86075-fig2-data1.zip › Figure 2-source data/F/raw unedited gels or blots/Myc with short time exposure.tif]

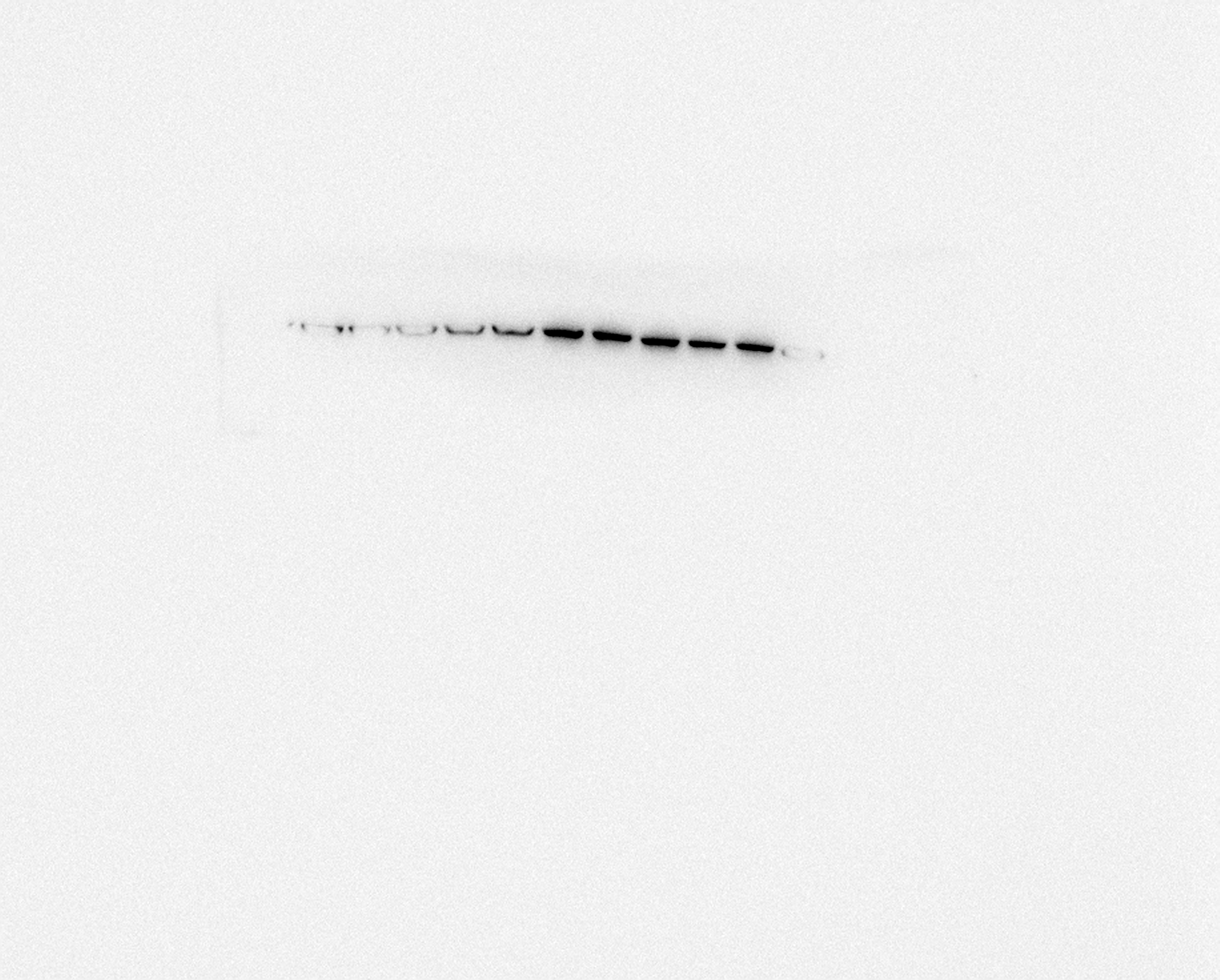

Supplement: Figure 2—source data 1. [file elife-86075-fig2-data1.zip › Figure 2-source data/C/raw unedited gels or blots/Tubulin.tif]

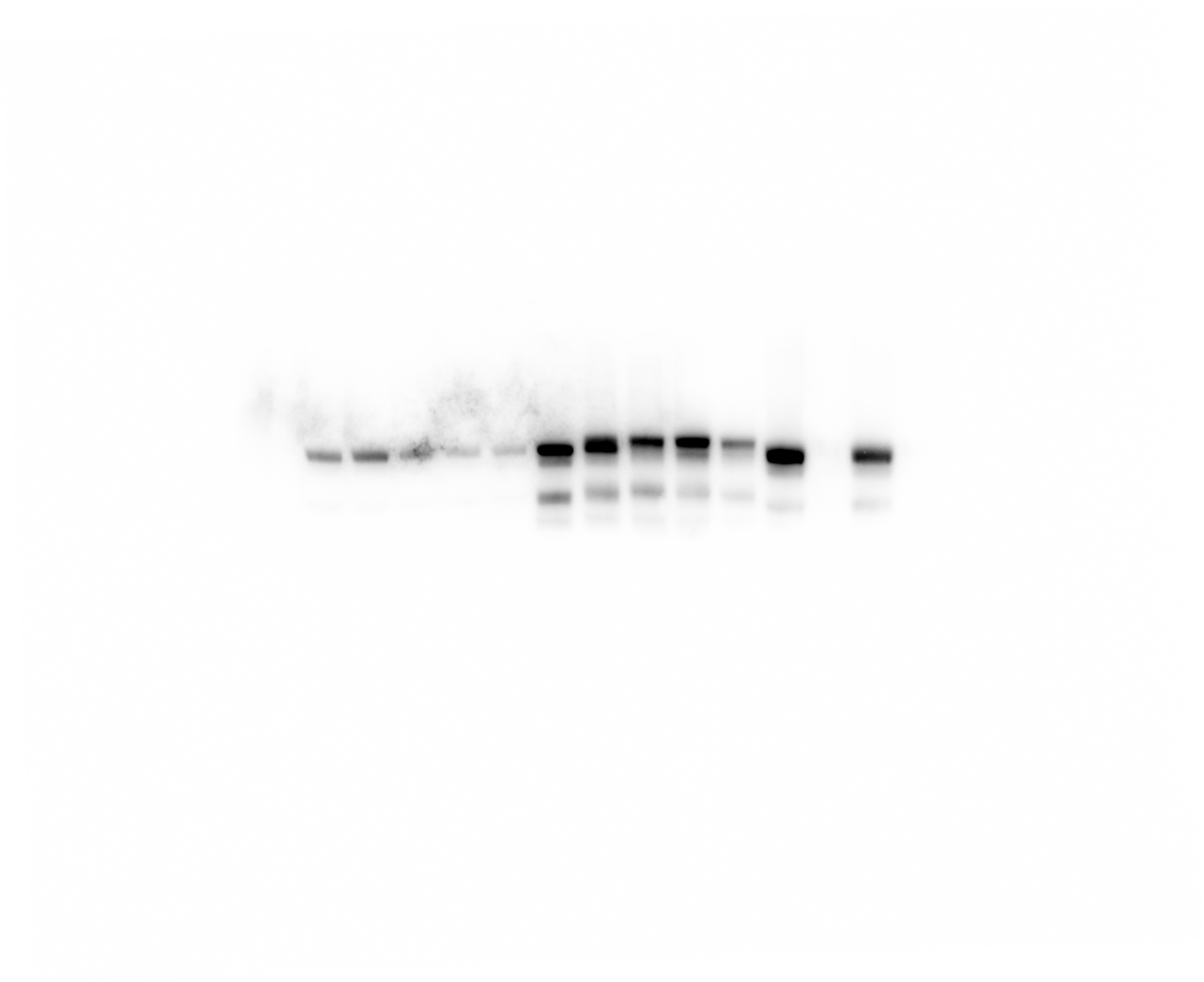

Supplement: Figure 2—source data 1. [file elife-86075-fig2-data1.zip › Figure 2-source data/C/raw unedited gels or blots/Myc.tif]

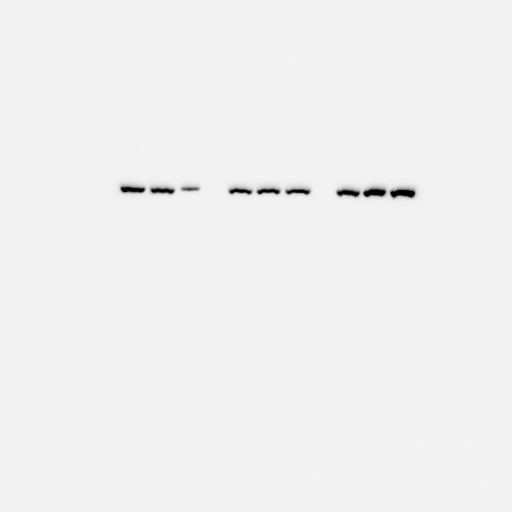

Supplement: Figure 2—source data 1. [file elife-86075-fig2-data1.zip › Figure 2-source data/E/raw unedited gels or blots/Tubulin.tif]

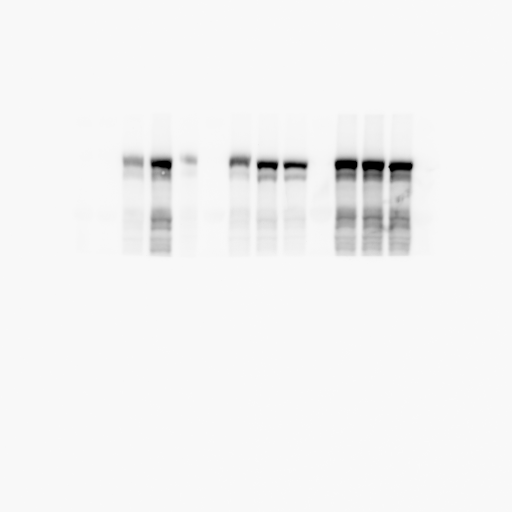

Supplement: Figure 2—source data 1. [file elife-86075-fig2-data1.zip › Figure 2-source data/E/raw unedited gels or blots/Myc.tif]

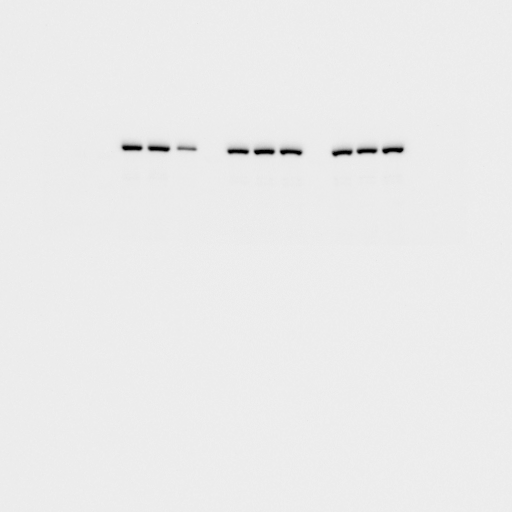

Supplement: Figure 2—source data 1. [file elife-86075-fig2-data1.zip › Figure 2-source data/B/raw unedited gels or blots/Tubulin.tif]

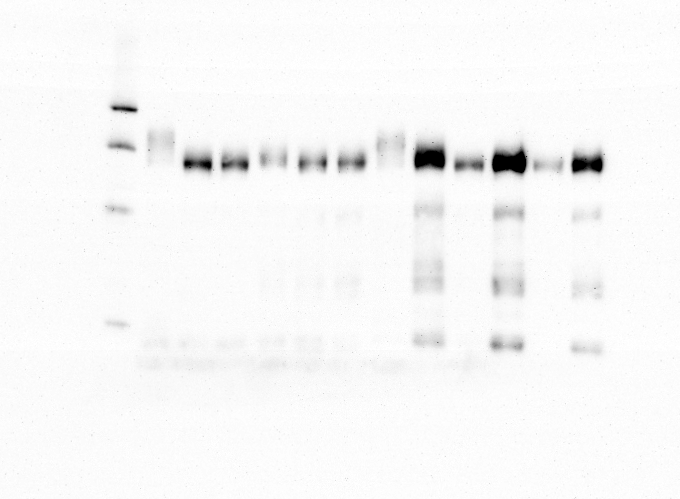

Supplement: Figure 2—source data 1. [file elife-86075-fig2-data1.zip › Figure 2-source data/B/raw unedited gels or blots/Myc.tif]

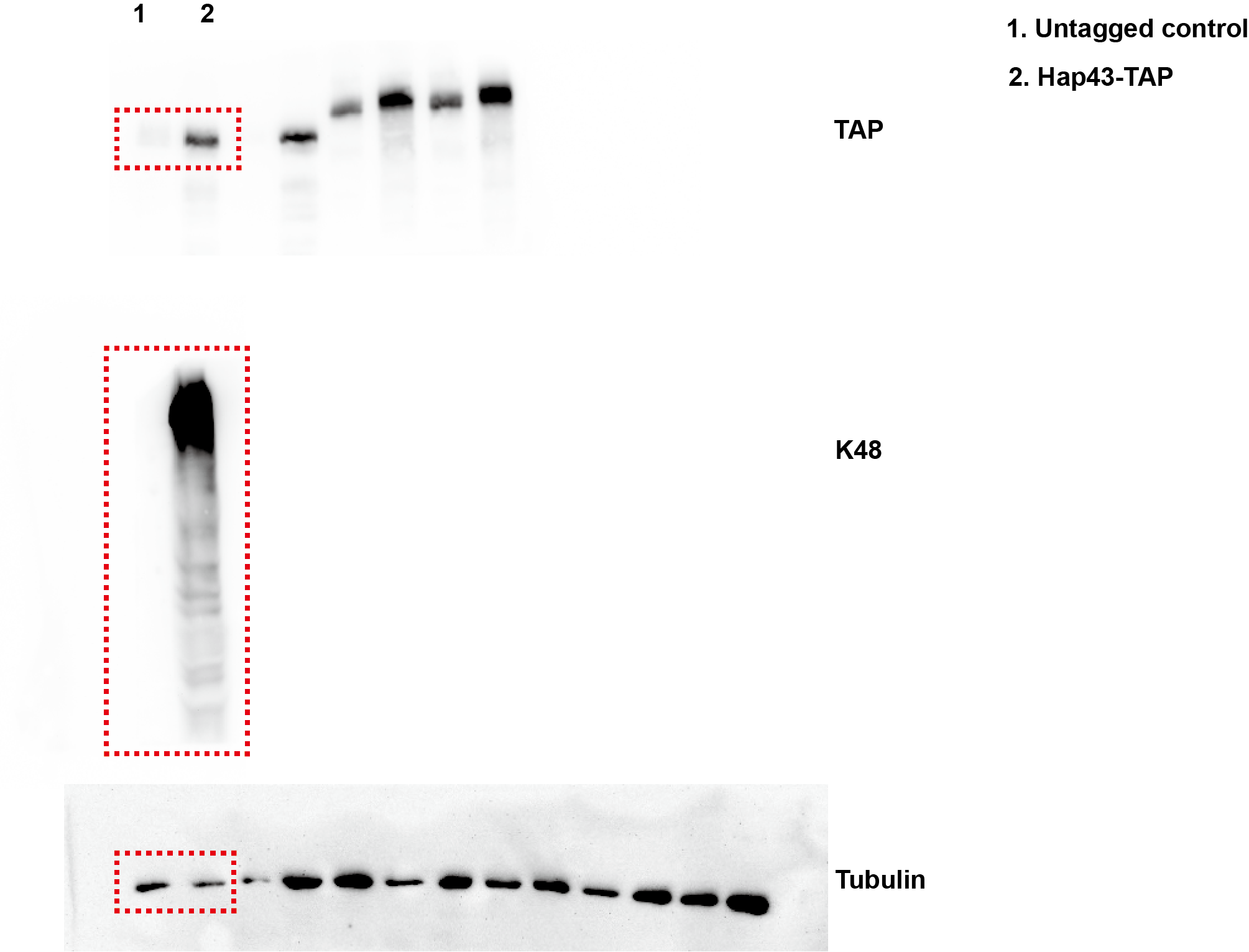

Supplement: Figure 3—source data 1. [file elife-86075-fig3-data1.zip › Figure 3-source data/G/Figure 3G with uncropped gels or blots.tif]

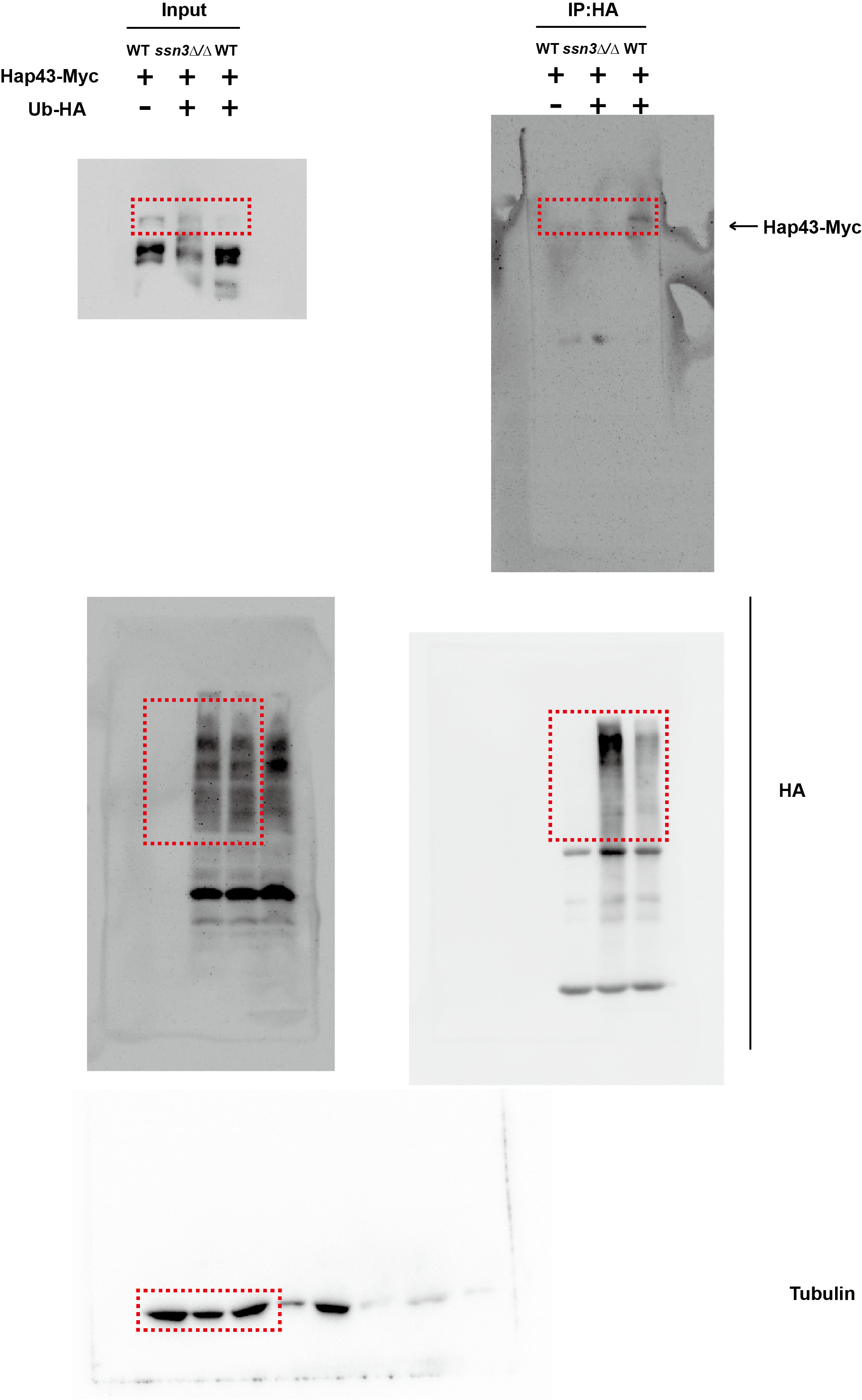

Supplement: Figure 3—source data 1. [file elife-86075-fig3-data1.zip › Figure 3-source data/F/Figure 3F with uncropped gels or blots.tif]

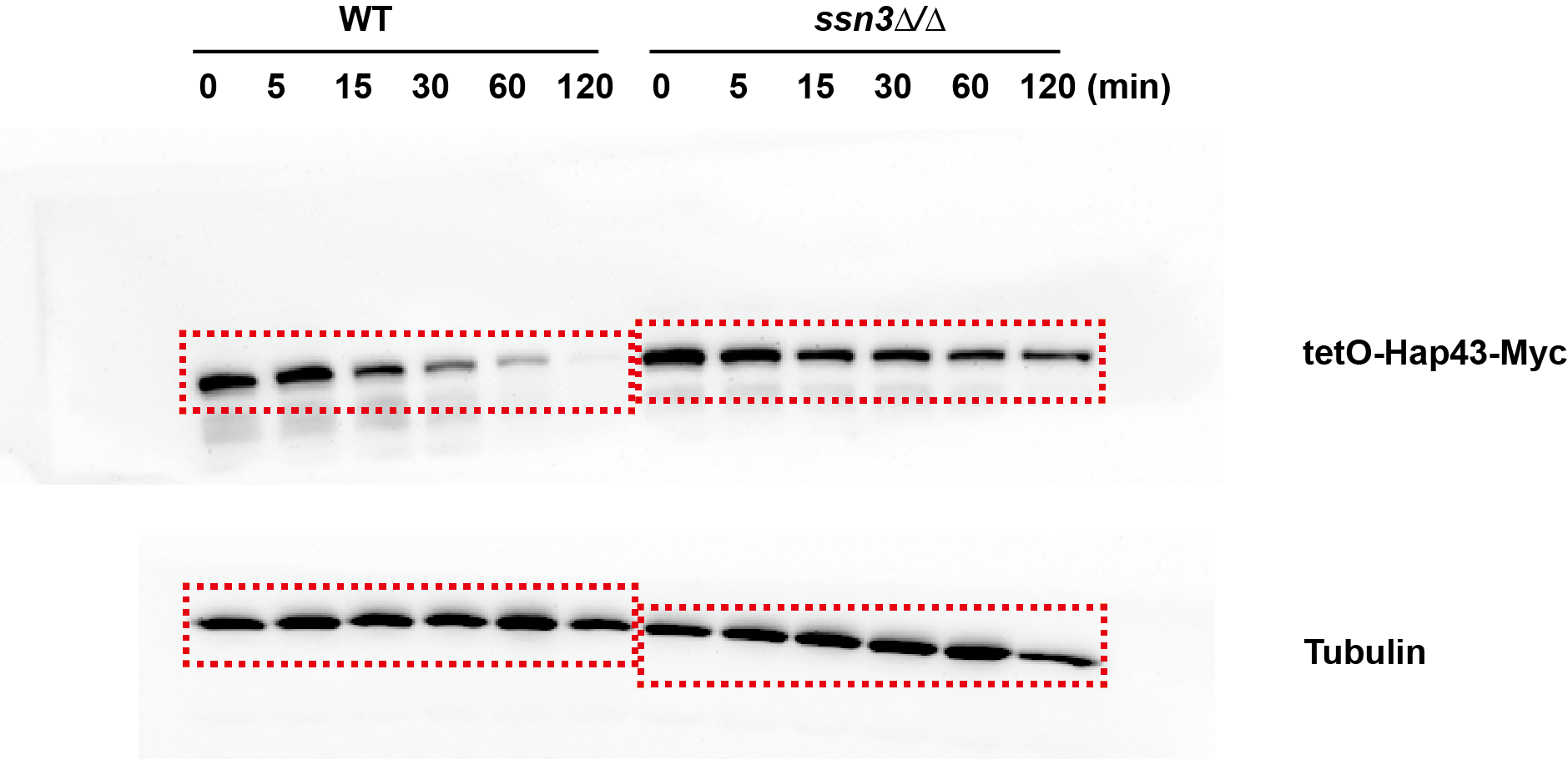

Supplement: Figure 3—source data 1. [file elife-86075-fig3-data1.zip › Figure 3-source data/D/Figure 3D with uncropped gels or blots.tif]

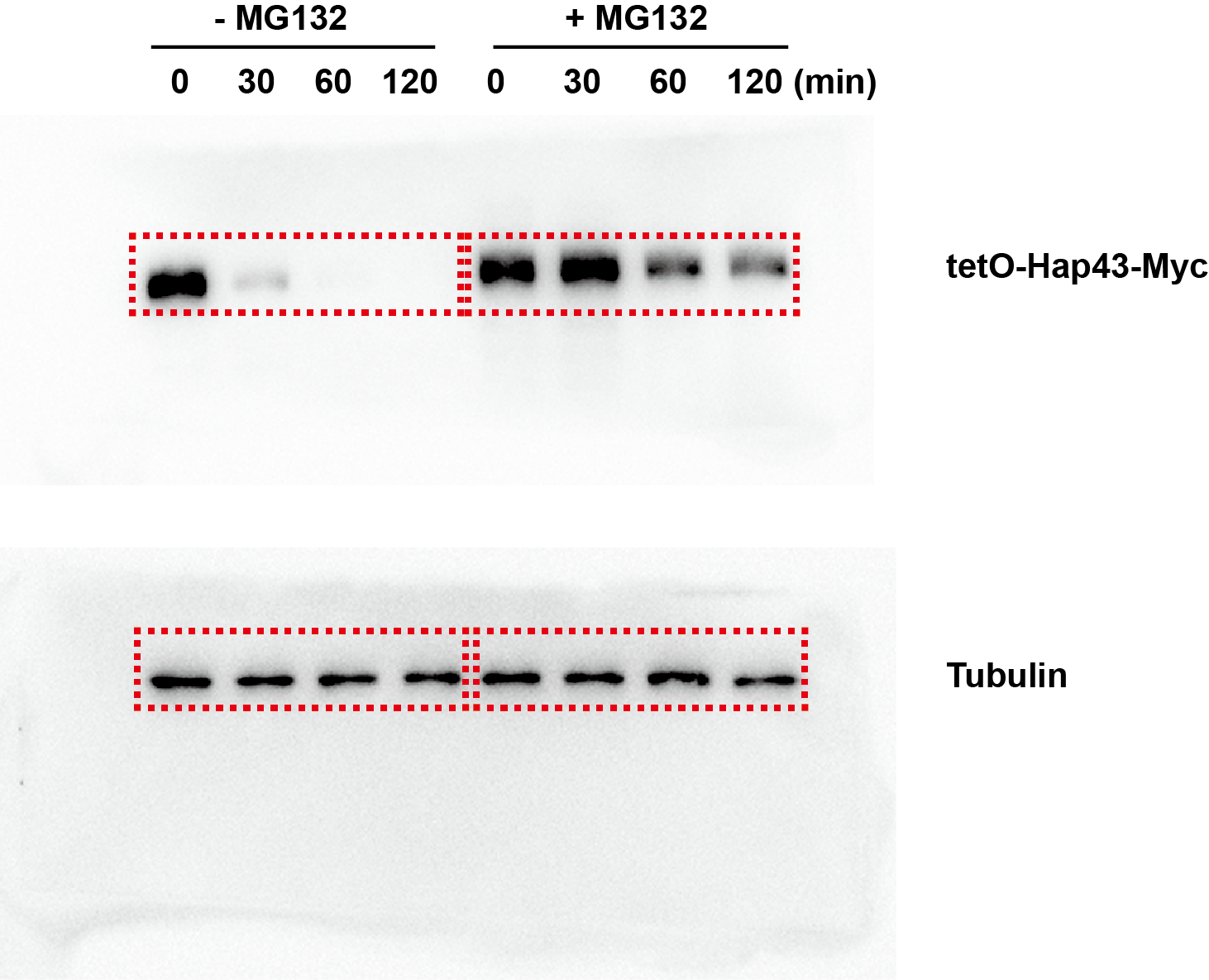

Supplement: Figure 3—source data 1. [file elife-86075-fig3-data1.zip › Figure 3-source data/E/Figure 3E with uncropped gels or blots.tif]

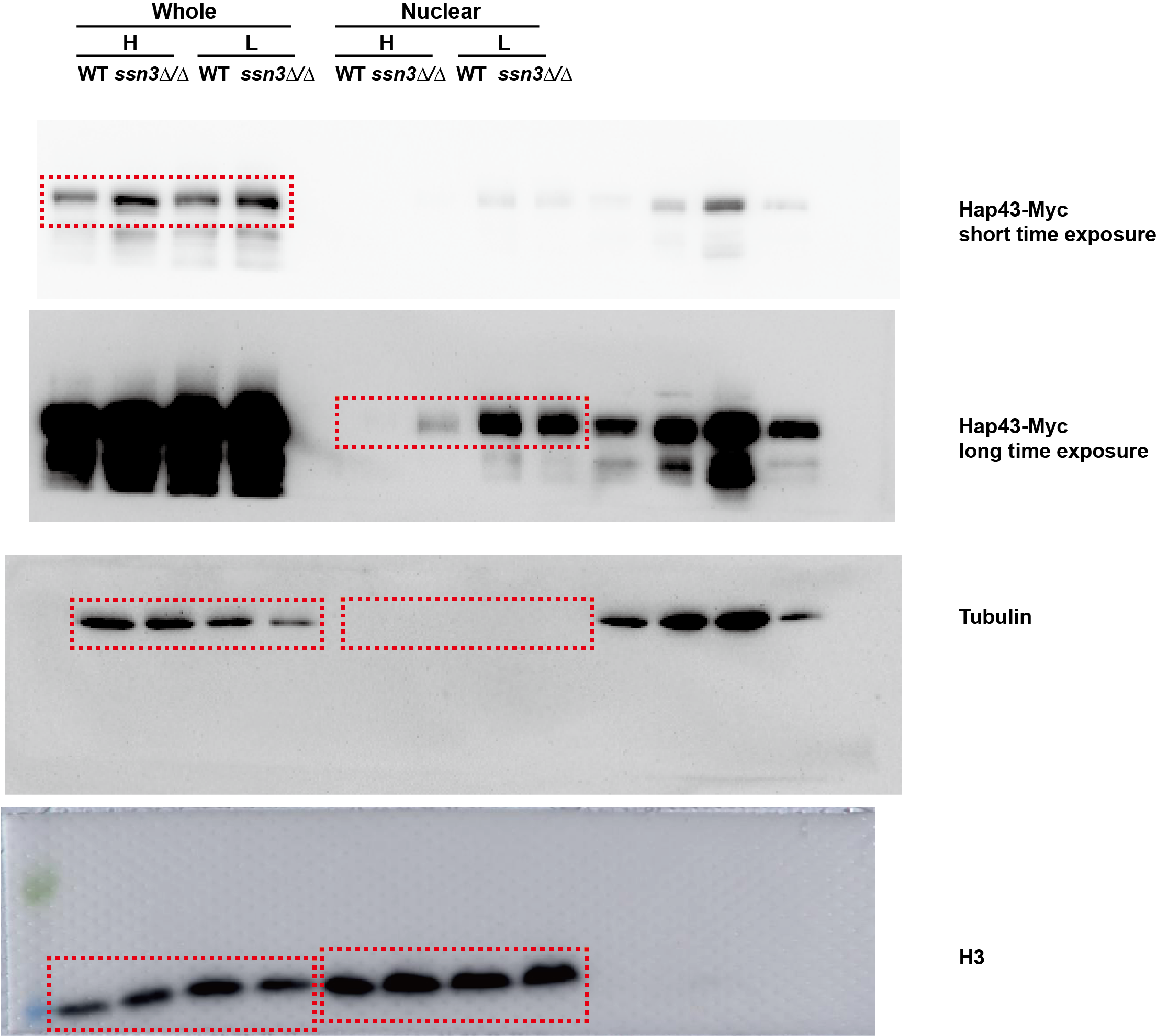

Supplement: Figure 3—source data 1. [file elife-86075-fig3-data1.zip › Figure 3-source data/B/Figure 3B with uncropped gels or blots.tif]

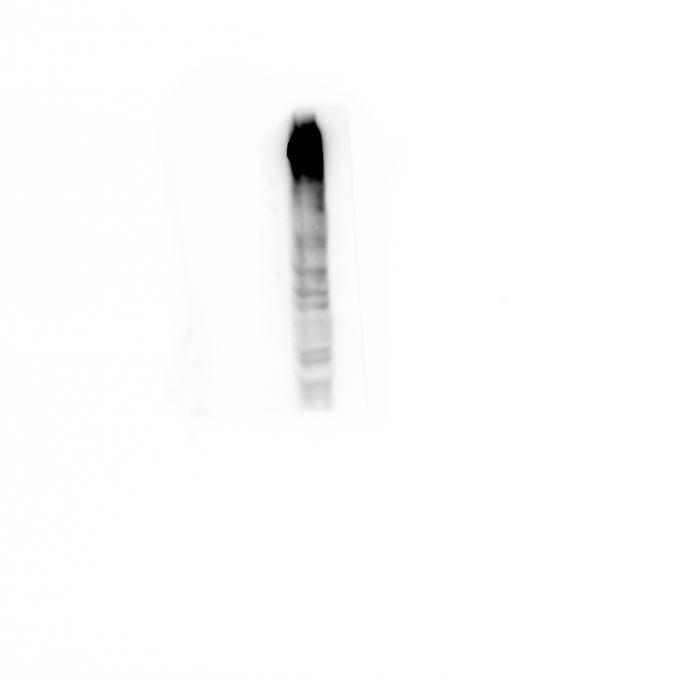

Supplement: Figure 3—source data 1. [file elife-86075-fig3-data1.zip › Figure 3-source data/G/raw unedited gels or blots/K48.tif]

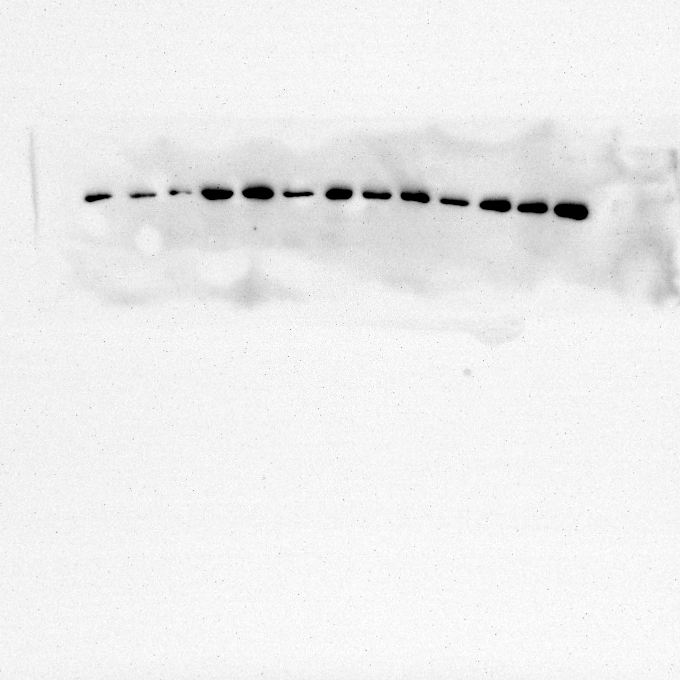

Supplement: Figure 3—source data 1. [file elife-86075-fig3-data1.zip › Figure 3-source data/G/raw unedited gels or blots/Tubulin.tif]

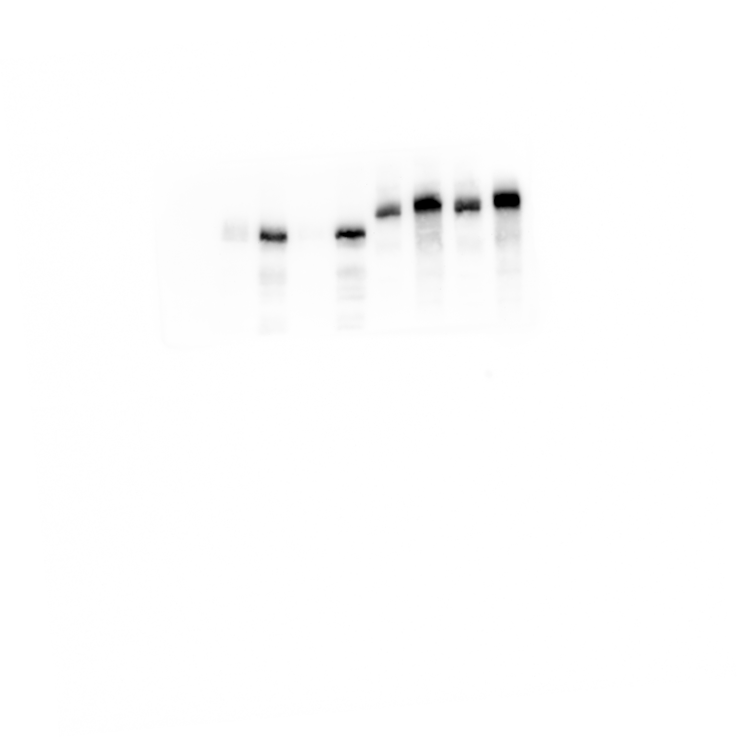

Supplement: Figure 3—source data 1. [file elife-86075-fig3-data1.zip › Figure 3-source data/G/raw unedited gels or blots/TAP.tif]

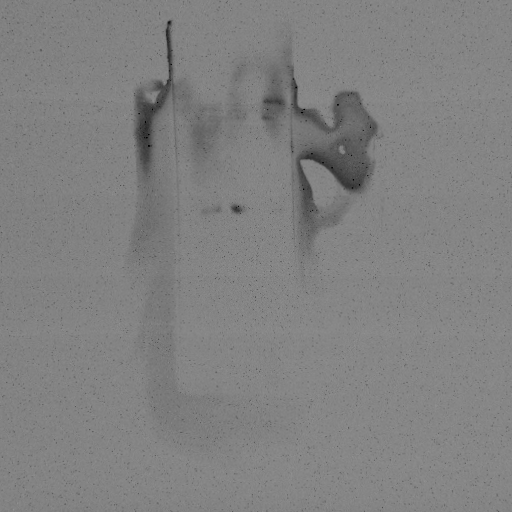

Supplement: Figure 3—source data 1. [file elife-86075-fig3-data1.zip › Figure 3-source data/F/raw unedited gels or blots/Myc IP.tif]

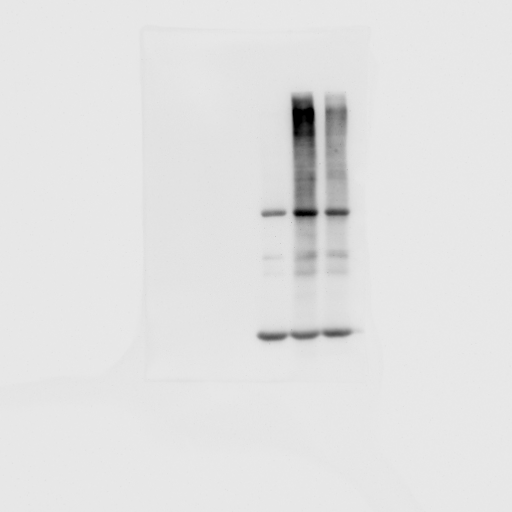

Supplement: Figure 3—source data 1. [file elife-86075-fig3-data1.zip › Figure 3-source data/F/raw unedited gels or blots/HA IP.tif]

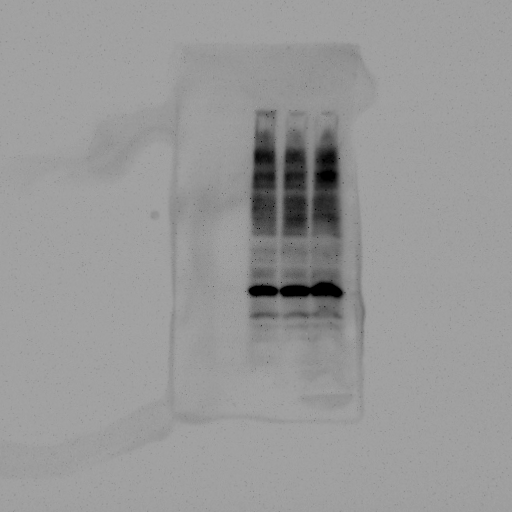

Supplement: Figure 3—source data 1. [file elife-86075-fig3-data1.zip › Figure 3-source data/F/raw unedited gels or blots/HA input.tif]

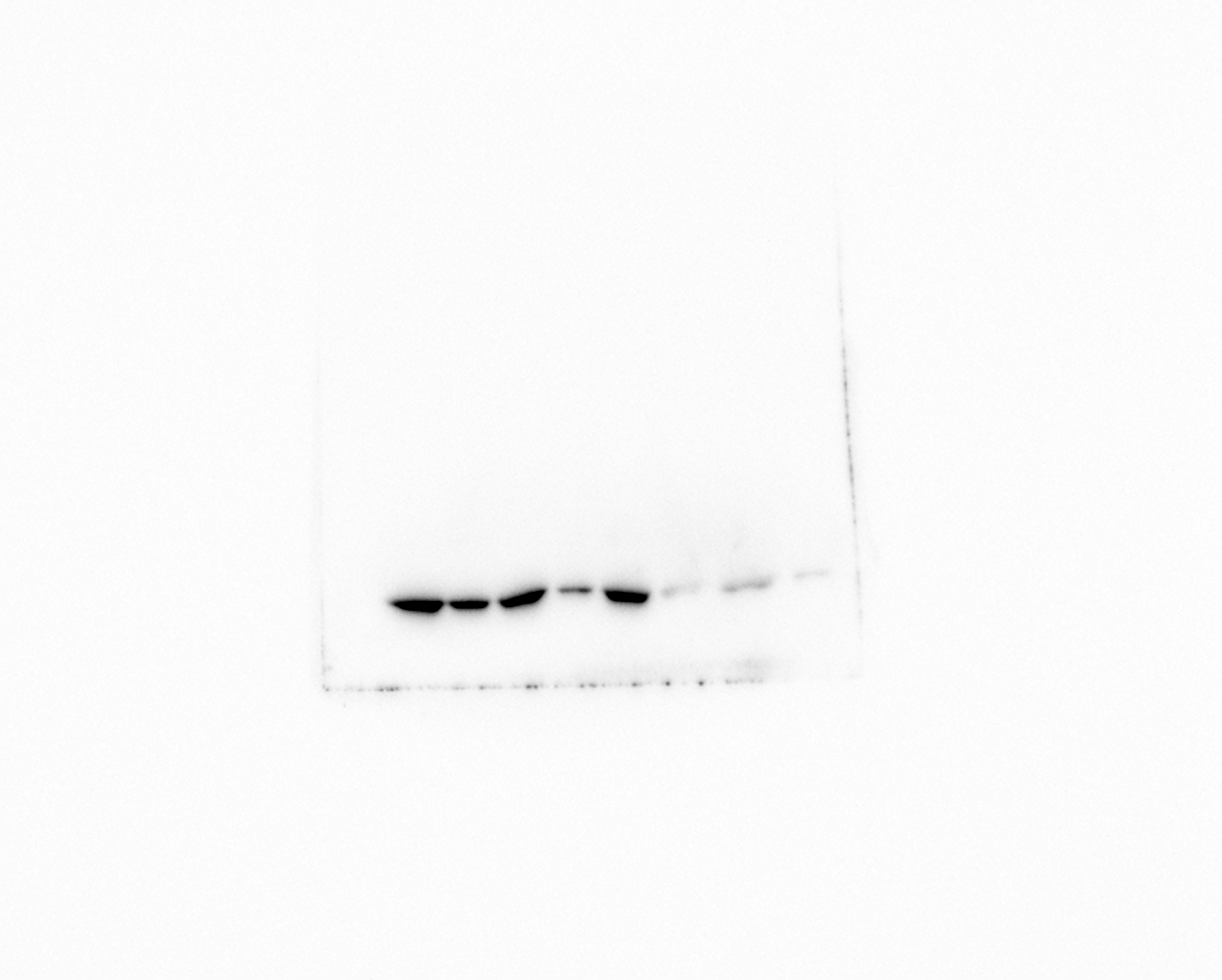

Supplement: Figure 3—source data 1. [file elife-86075-fig3-data1.zip › Figure 3-source data/F/raw unedited gels or blots/Tubulin.tif]

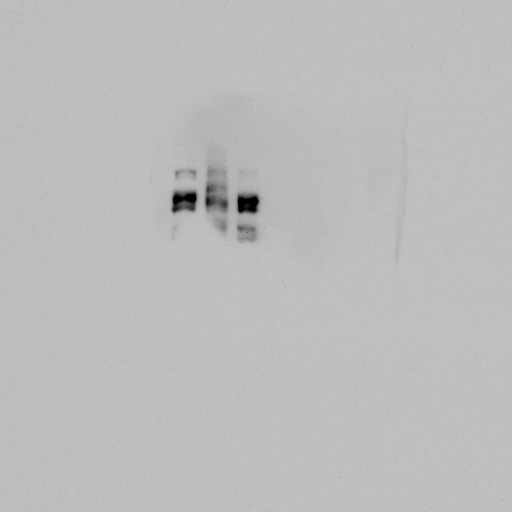

Supplement: Figure 3—source data 1. [file elife-86075-fig3-data1.zip › Figure 3-source data/F/raw unedited gels or blots/Myc input.tif]

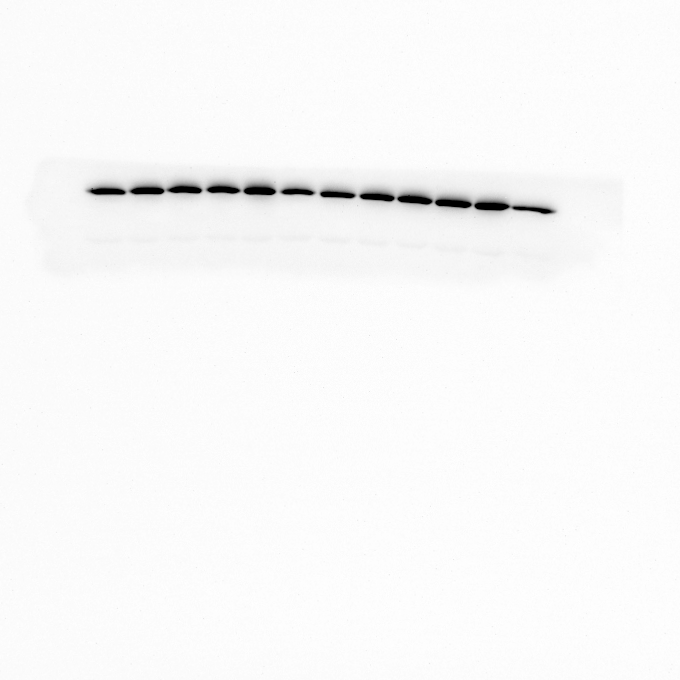

Supplement: Figure 3—source data 1. [file elife-86075-fig3-data1.zip › Figure 3-source data/D/raw unedited gels or blots/Tubulin.tif]

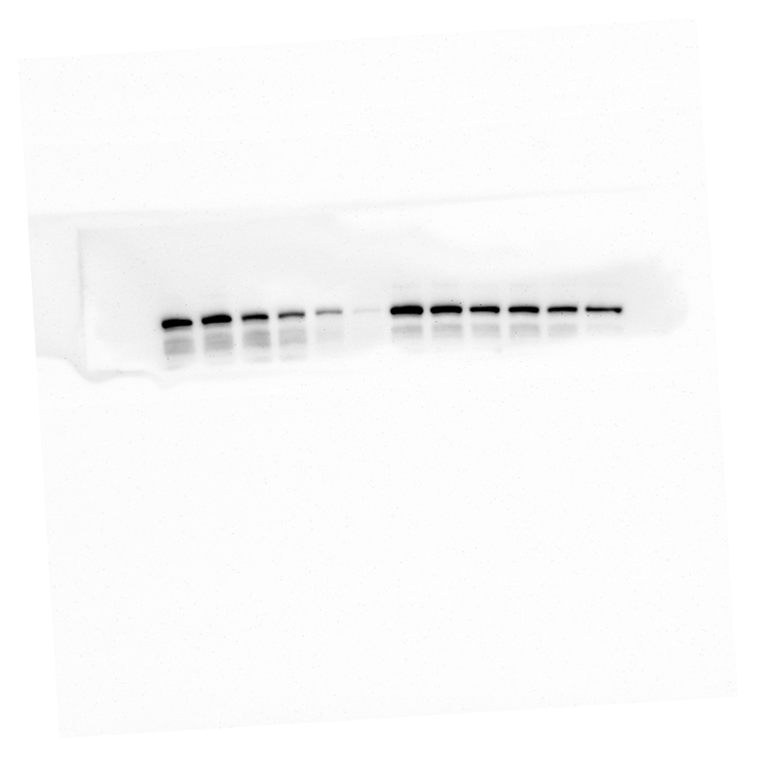

Supplement: Figure 3—source data 1. [file elife-86075-fig3-data1.zip › Figure 3-source data/D/raw unedited gels or blots/Myc.tif]

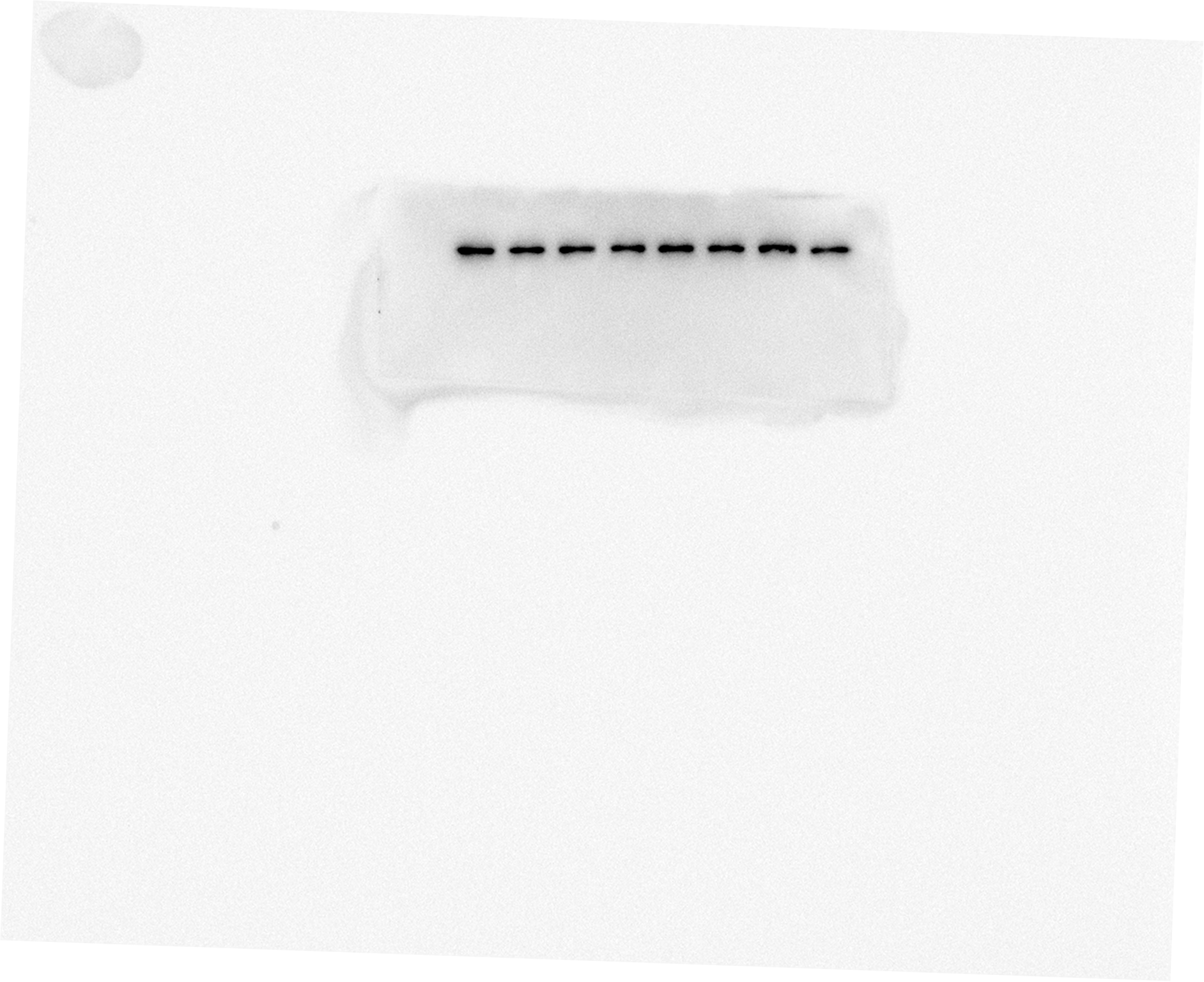

Supplement: Figure 3—source data 1. [file elife-86075-fig3-data1.zip › Figure 3-source data/E/raw unedited gels or blots/Tubulin.tif]

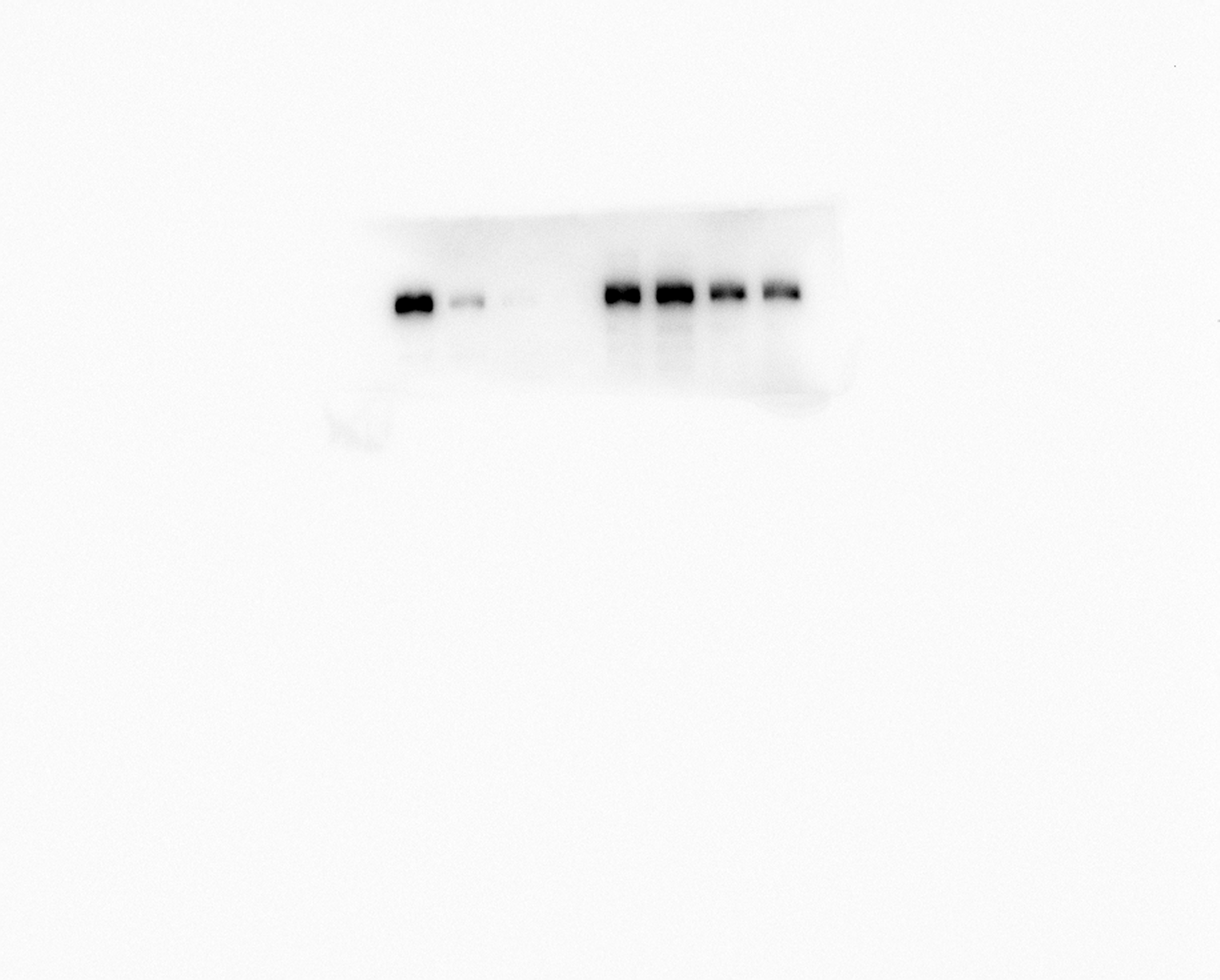

Supplement: Figure 3—source data 1. [file elife-86075-fig3-data1.zip › Figure 3-source data/E/raw unedited gels or blots/Myc.tif]

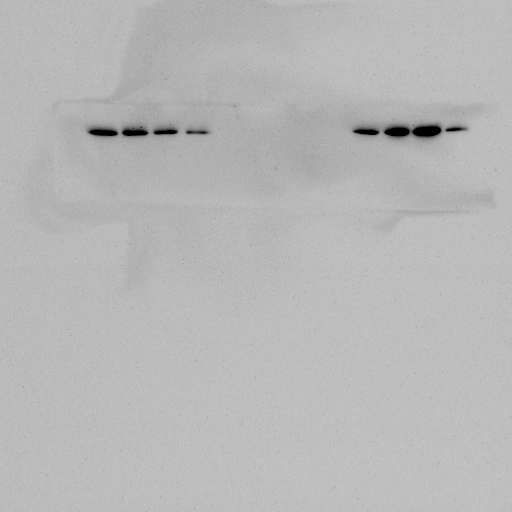

Supplement: Figure 3—source data 1. [file elife-86075-fig3-data1.zip › Figure 3-source data/B/raw unedited gels or blots/Tubulin.tif]

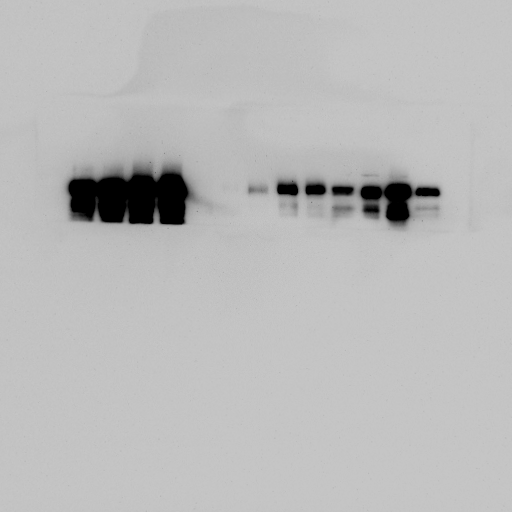

Supplement: Figure 3—source data 1. [file elife-86075-fig3-data1.zip › Figure 3-source data/B/raw unedited gels or blots/Myc with long time exposure.tif]

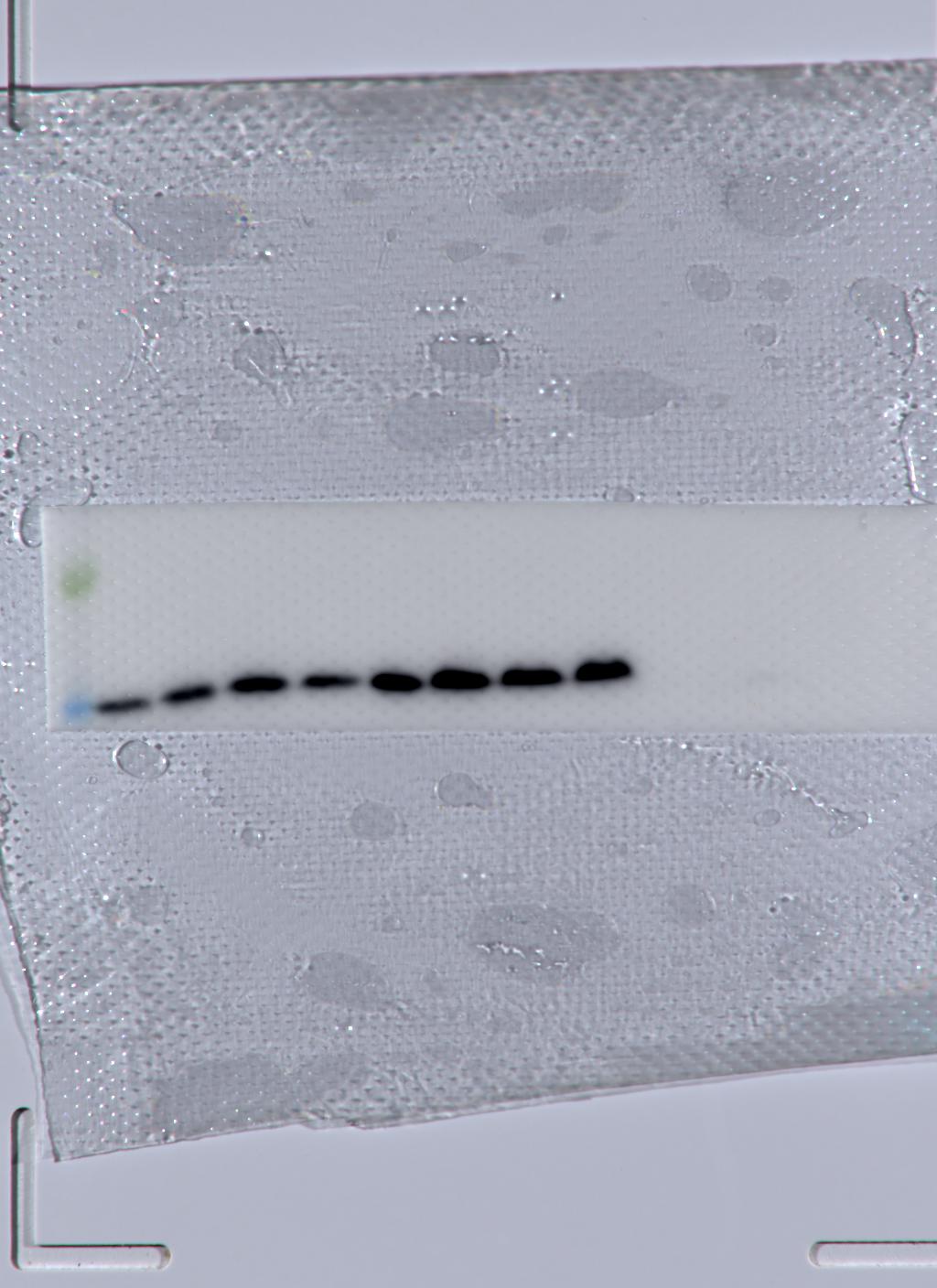

Supplement: Figure 3—source data 1. [file elife-86075-fig3-data1.zip › Figure 3-source data/B/raw unedited gels or blots/H3 .jpg]

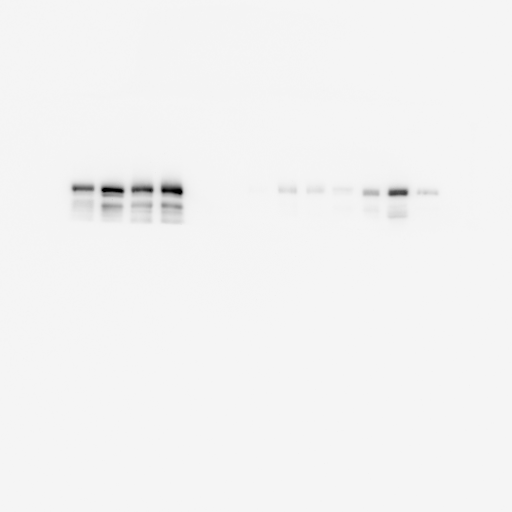

Supplement: Figure 3—source data 1. [file elife-86075-fig3-data1.zip › Figure 3-source data/B/raw unedited gels or blots/Myc with short time exposure.tif]

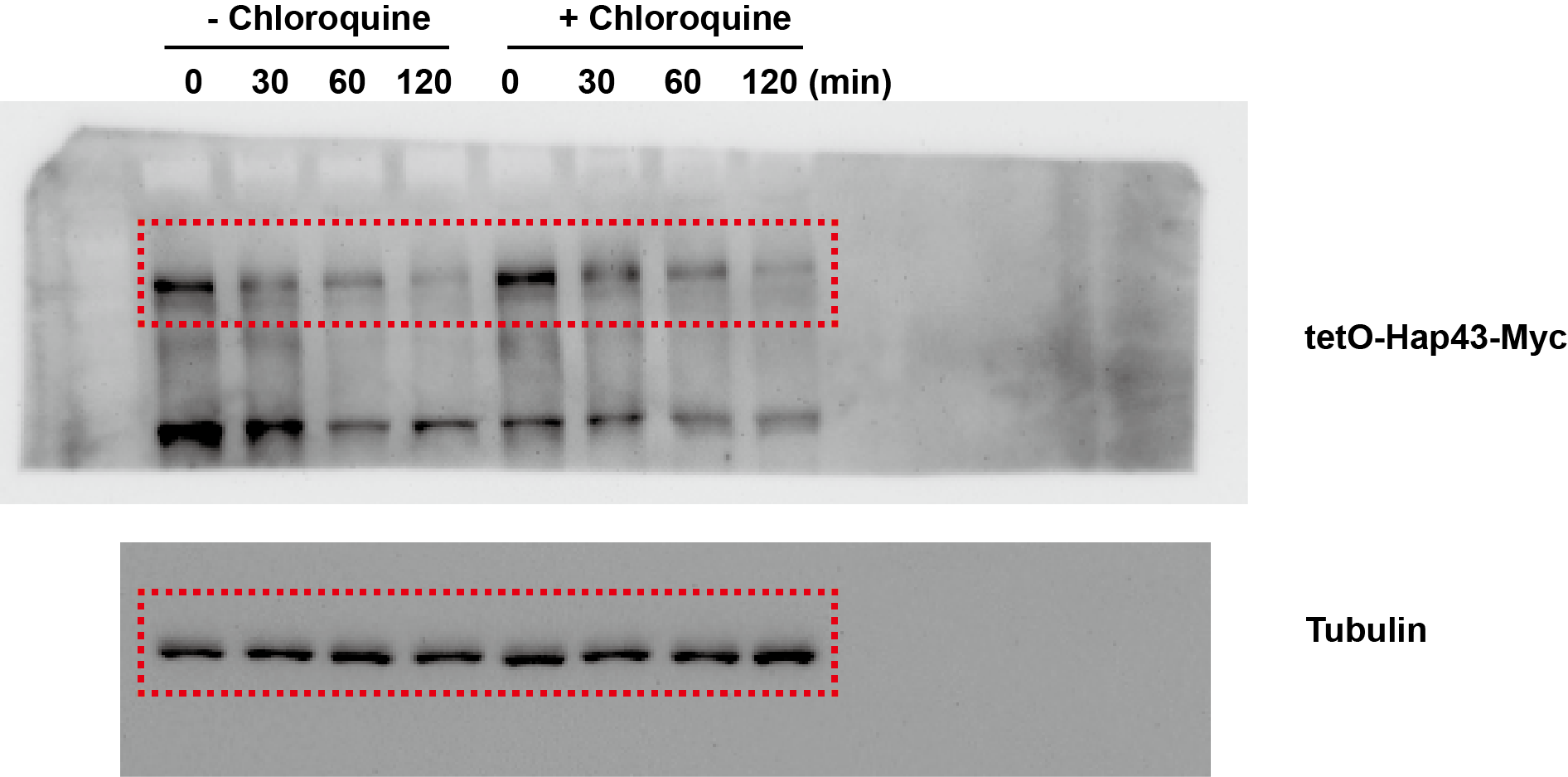

Supplement: Figure 3—figure supplement 1—source data 1. [file elife-86075-fig3-figsupp1-data1.zip › Figure 3ΓÇöfigure supplement 1-source data/Figure 3ΓÇöfigure supplement 1 with uncropped gels or blots.tif]

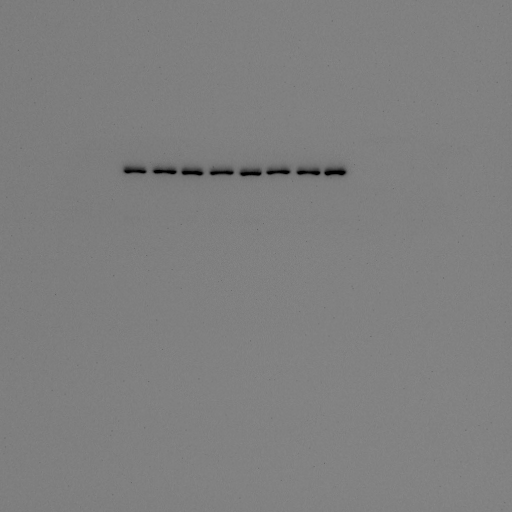

Supplement: Figure 3—figure supplement 1—source data 1. [file elife-86075-fig3-figsupp1-data1.zip › Figure 3ΓÇöfigure supplement 1-source data/raw unedited gels or blots/Tubulin.tif]

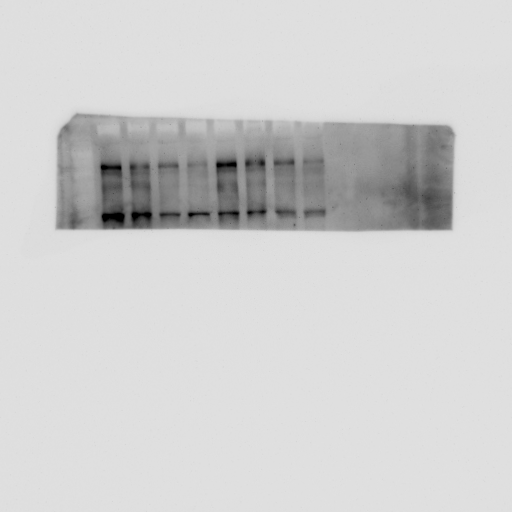

Supplement: Figure 3—figure supplement 1—source data 1. [file elife-86075-fig3-figsupp1-data1.zip › Figure 3ΓÇöfigure supplement 1-source data/raw unedited gels or blots/Myc.tif]

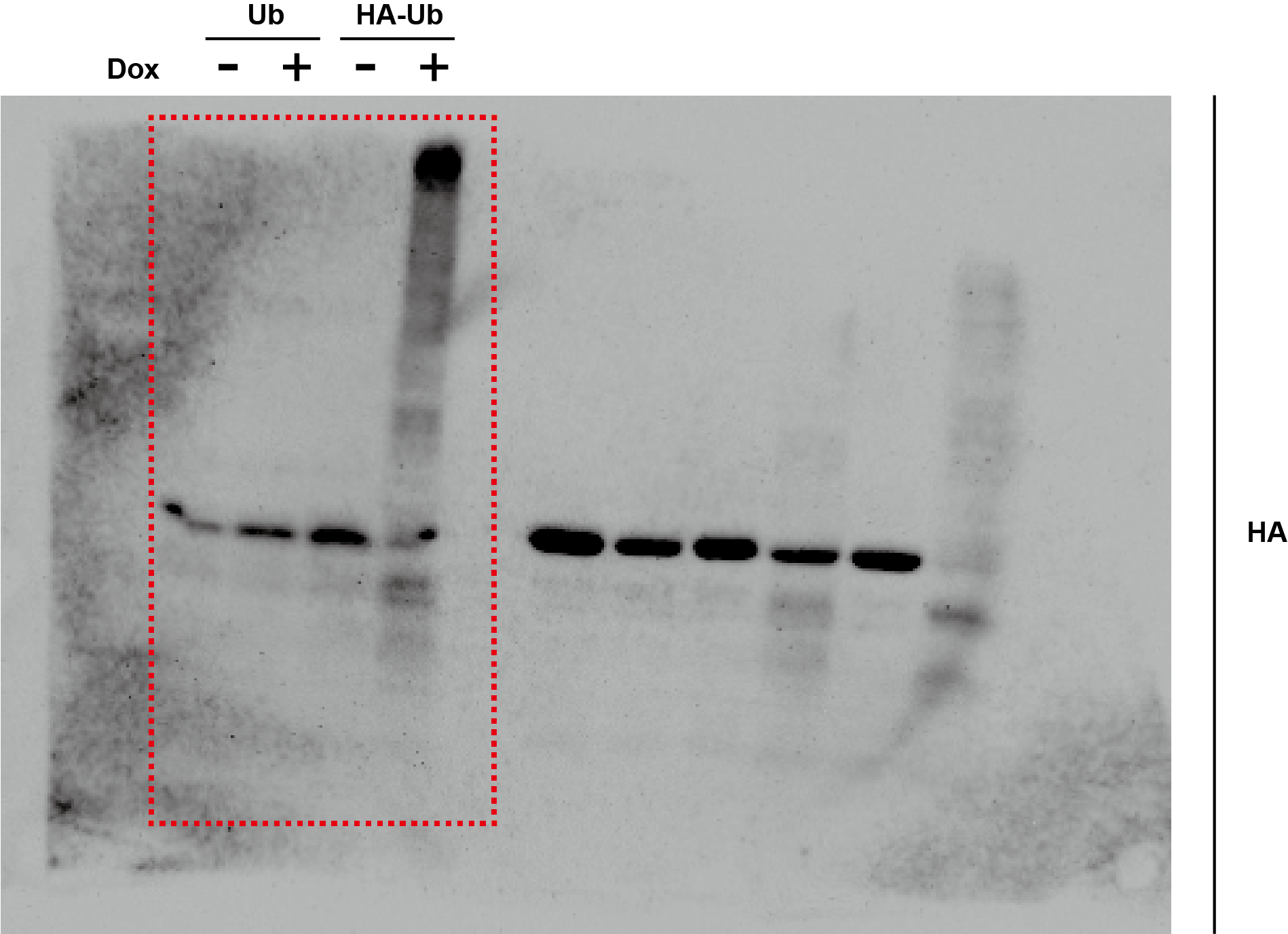

Supplement: Figure 3—figure supplement 2—source data 1. [file elife-86075-fig3-figsupp2-data1.zip › Figure 3ΓÇöfigure supplement 2-source data/Figure 3ΓÇöfigure supplement 2 with uncropped gels or blots.tif]

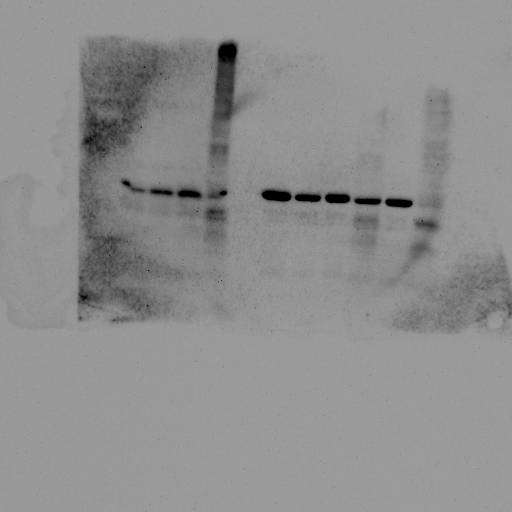

Supplement: Figure 3—figure supplement 2—source data 1. [file elife-86075-fig3-figsupp2-data1.zip › Figure 3ΓÇöfigure supplement 2-source data/raw unedited gels or blots/HA.tif]

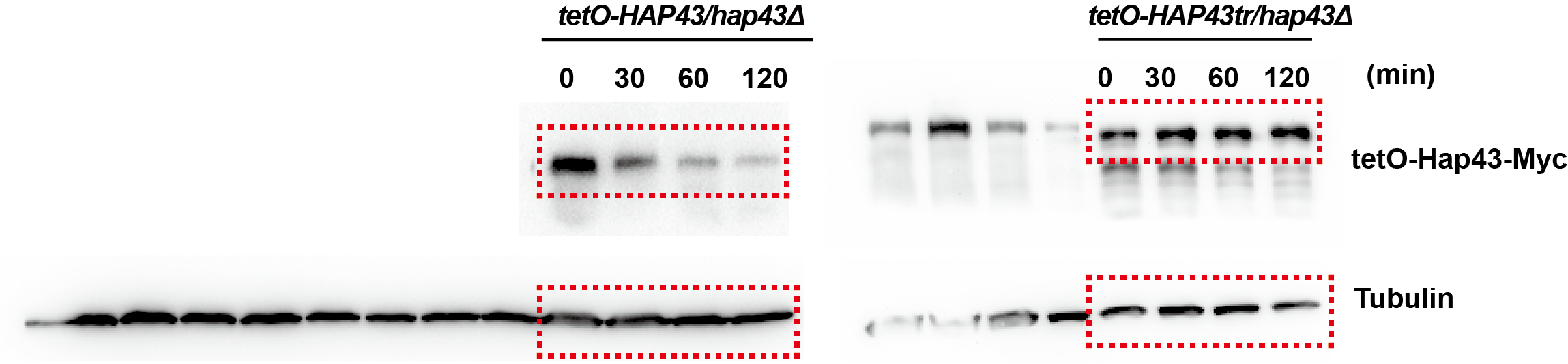

Supplement: Figure 4—source data 1. [file elife-86075-fig4-data1.zip › Figure 4-source data/F/Figure 4F with uncropped gels or blots.tif]

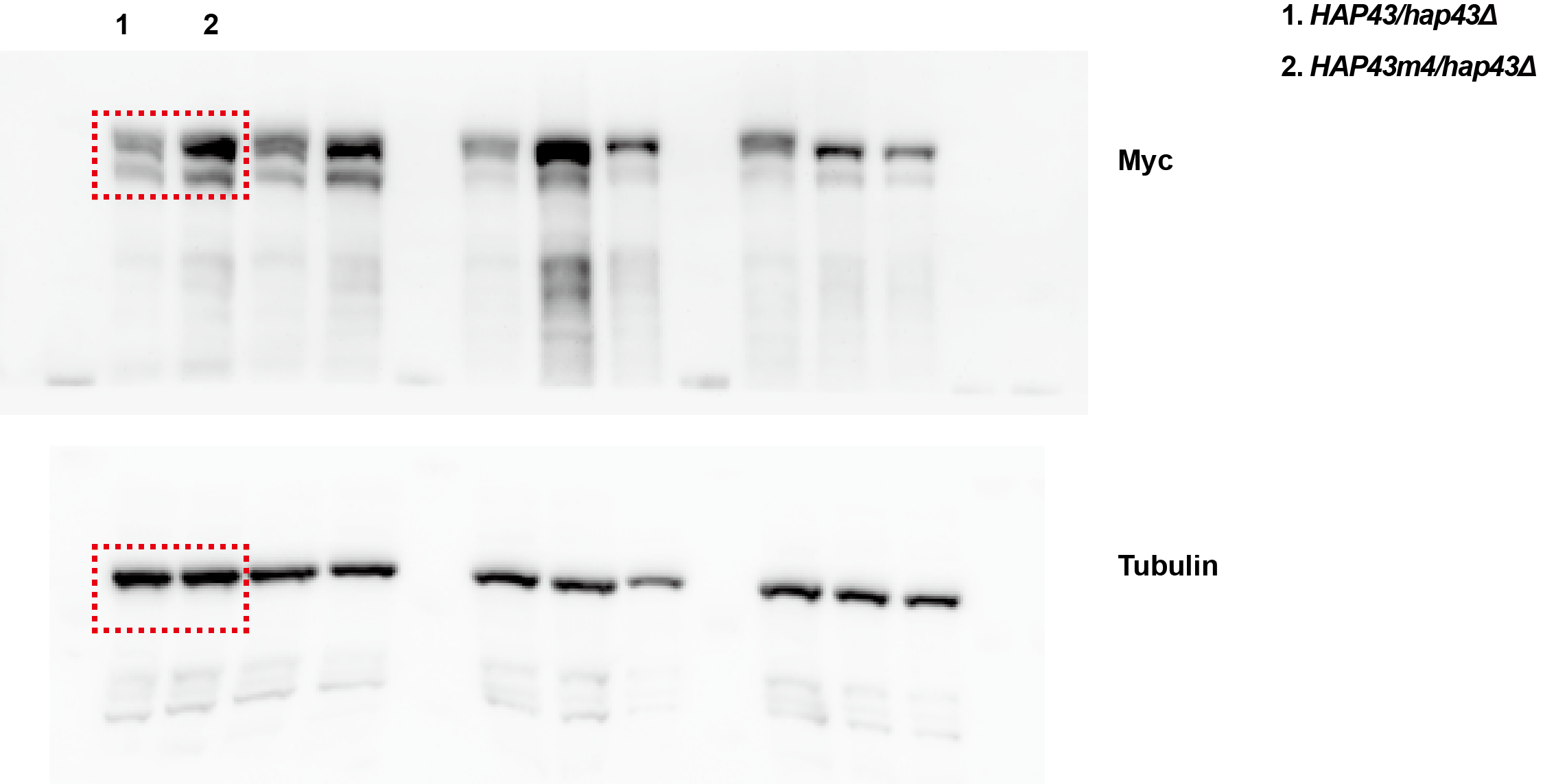

Supplement: Figure 4—source data 1. [file elife-86075-fig4-data1.zip › Figure 4-source data/H/Figure 4H with uncropped gels or blots.tif]

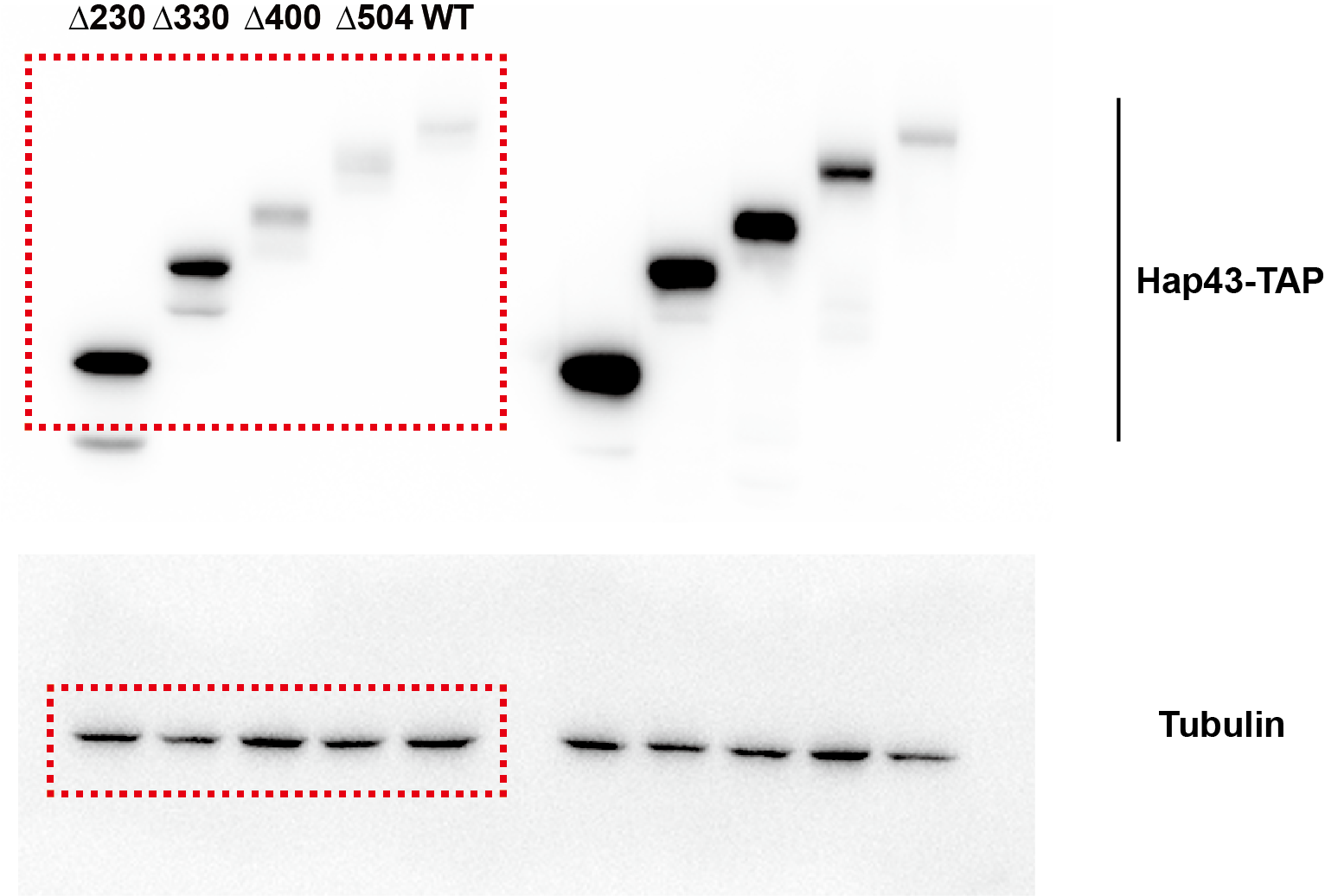

Supplement: Figure 4—source data 1. [file elife-86075-fig4-data1.zip › Figure 4-source data/C/Figure 4C with uncropped gels or blots.tif]

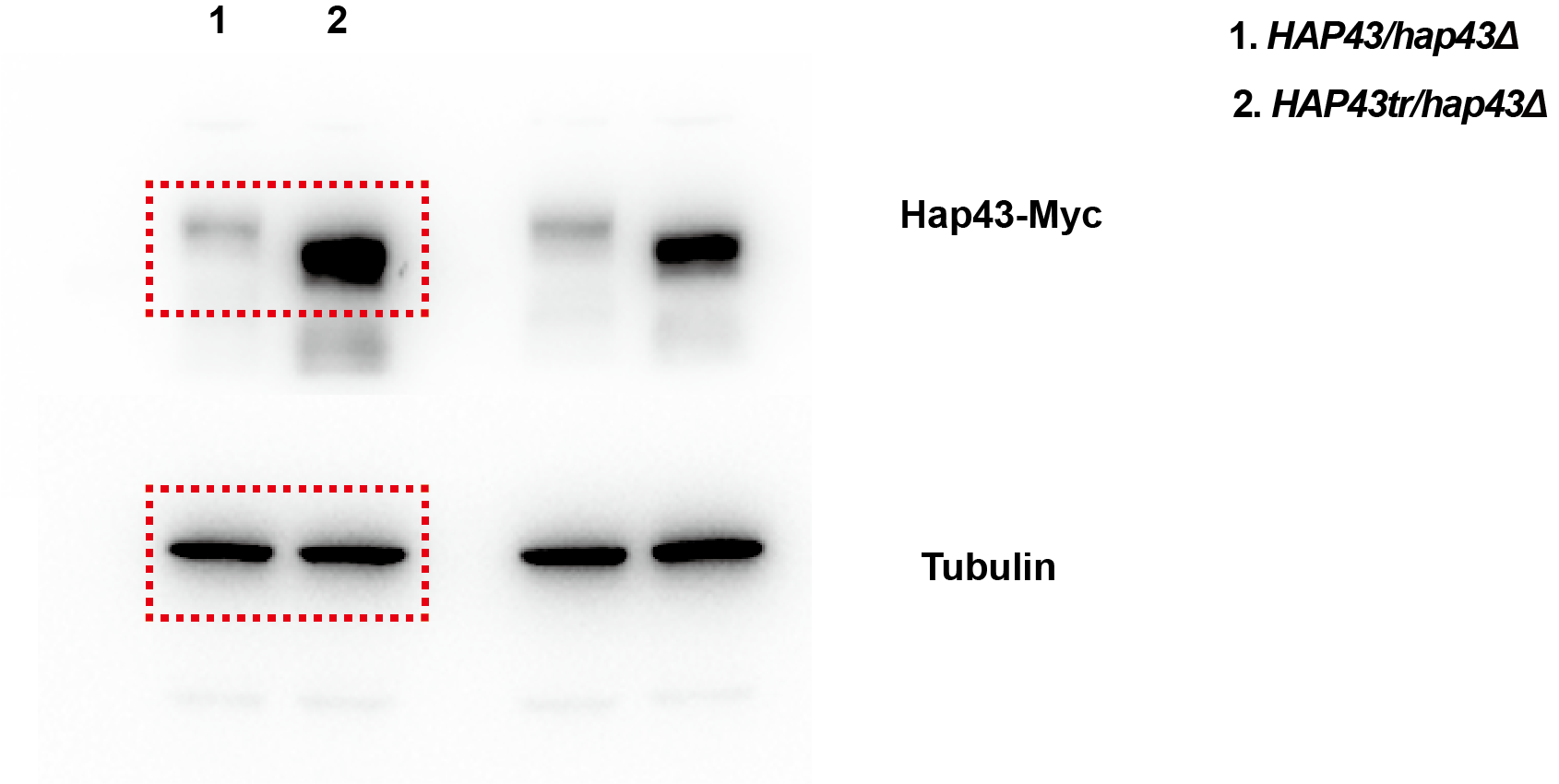

Supplement: Figure 4—source data 1. [file elife-86075-fig4-data1.zip › Figure 4-source data/E/Figure 4E with uncropped gels or blots.tif]

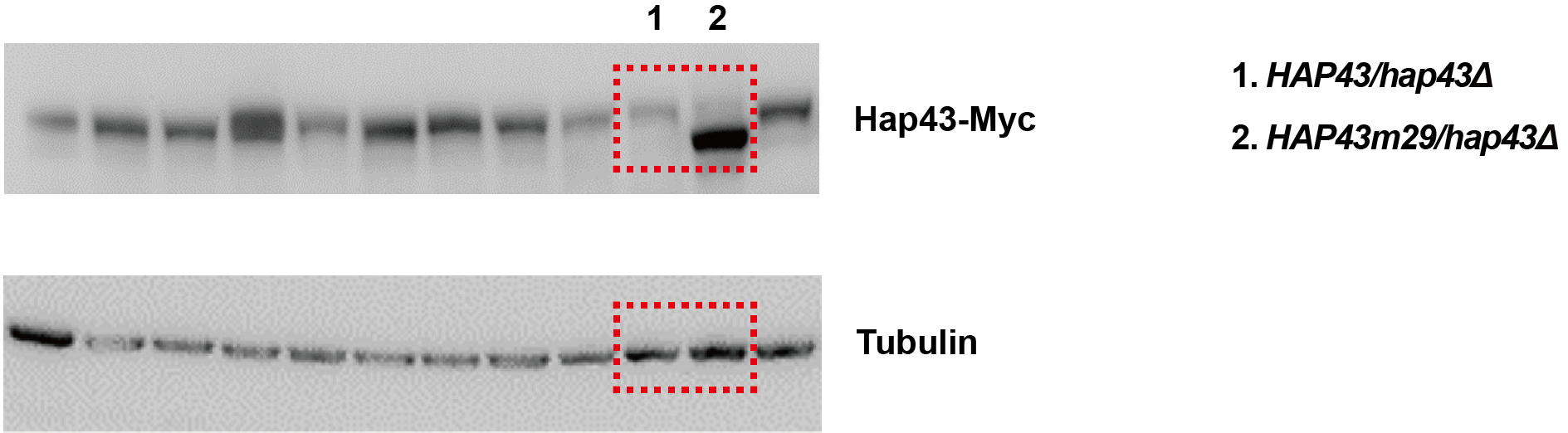

Supplement: Figure 4—source data 1. [file elife-86075-fig4-data1.zip › Figure 4-source data/B/Figure 4B with uncropped gels or blots.tif]

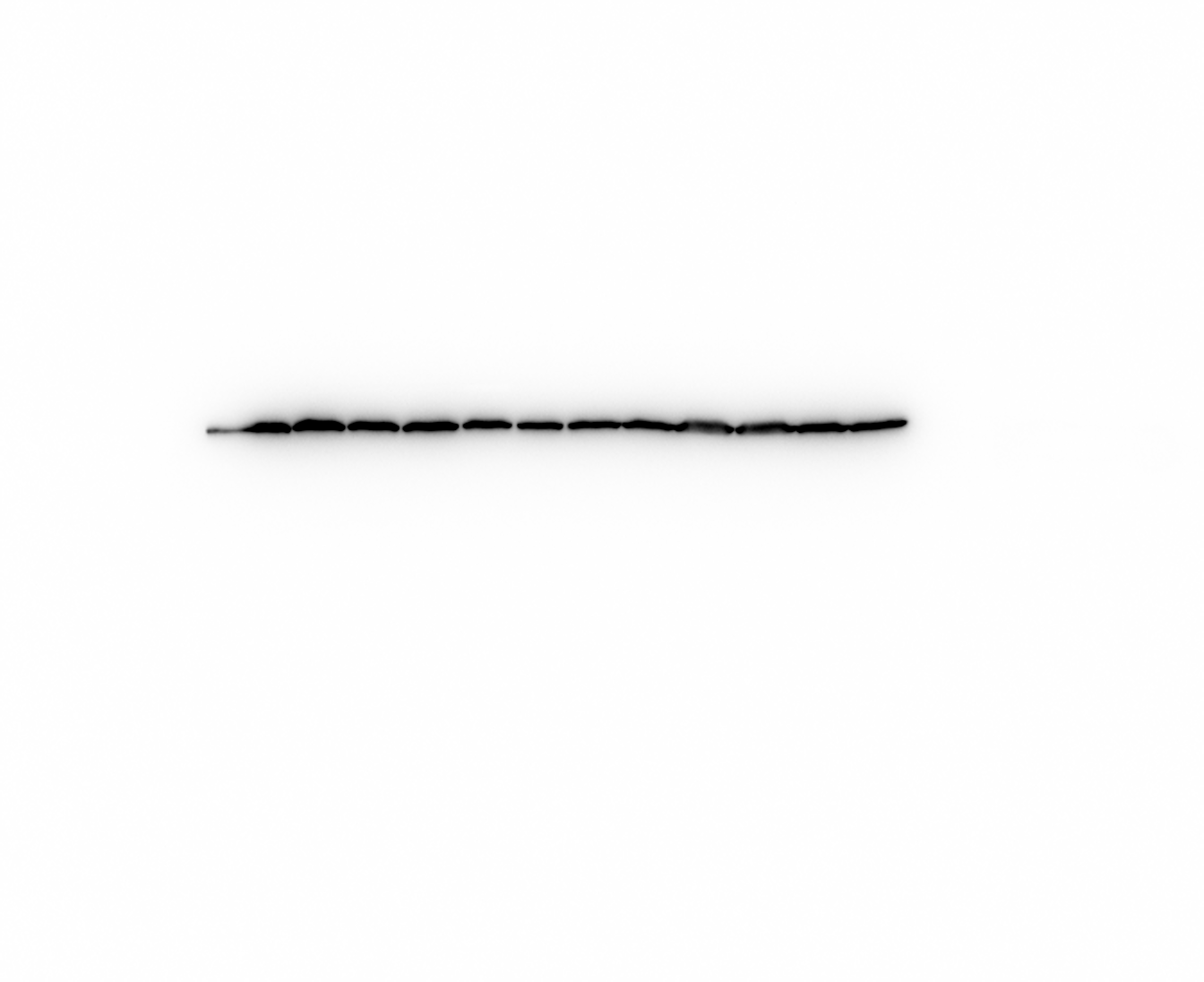

Supplement: Figure 4—source data 1. [file elife-86075-fig4-data1.zip › Figure 4-source data/F/raw unedited gels or blots/Tubulin_left panel.Tif]

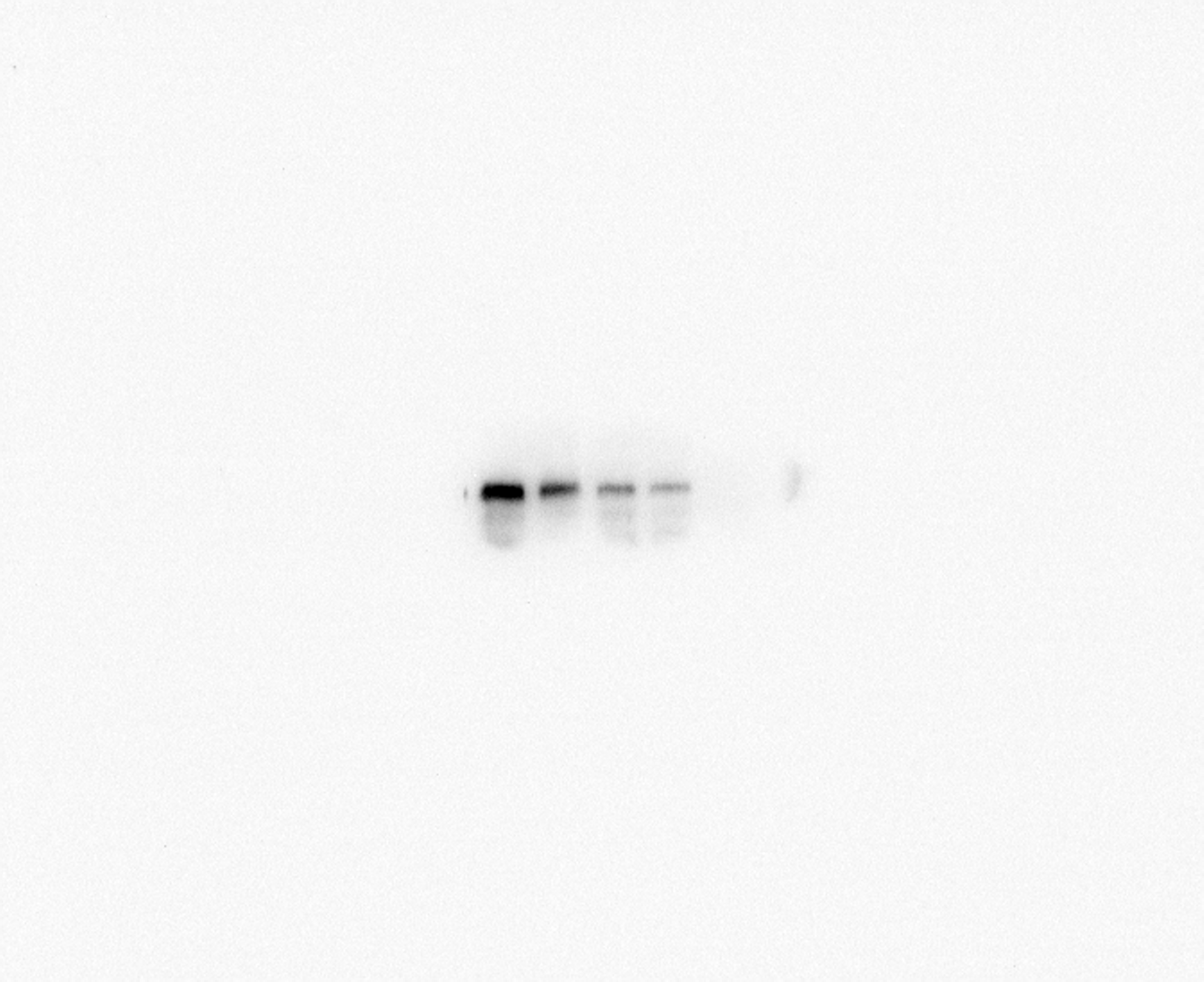

Supplement: Figure 4—source data 1. [file elife-86075-fig4-data1.zip › Figure 4-source data/F/raw unedited gels or blots/Myc_left panel.Tif]

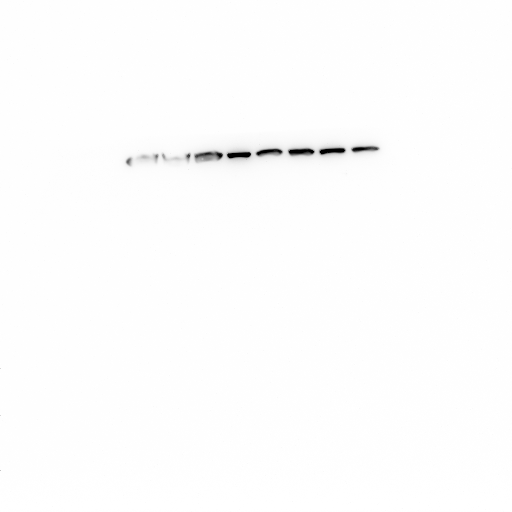

Supplement: Figure 4—source data 1. [file elife-86075-fig4-data1.zip › Figure 4-source data/F/raw unedited gels or blots/Tubulin_right panel.tif]

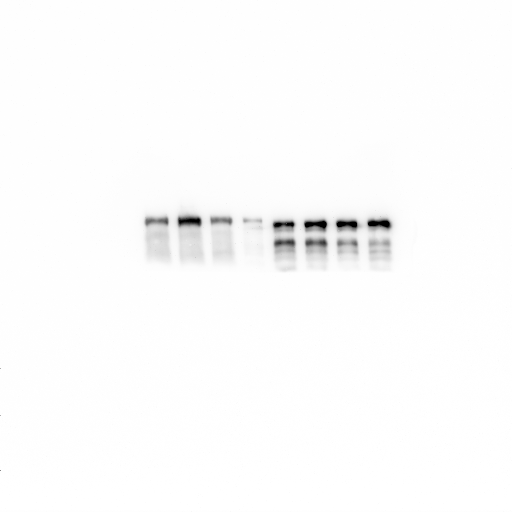

Supplement: Figure 4—source data 1. [file elife-86075-fig4-data1.zip › Figure 4-source data/F/raw unedited gels or blots/Myc_right panel.tif]

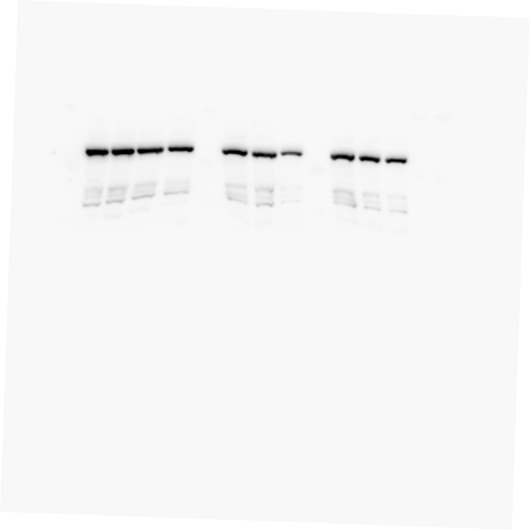

Supplement: Figure 4—source data 1. [file elife-86075-fig4-data1.zip › Figure 4-source data/H/raw unedited gels or blots/Tubulin.tif]

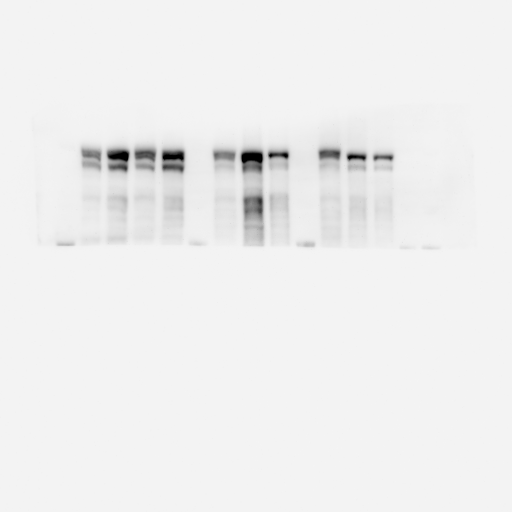

Supplement: Figure 4—source data 1. [file elife-86075-fig4-data1.zip › Figure 4-source data/H/raw unedited gels or blots/Myc.tif]

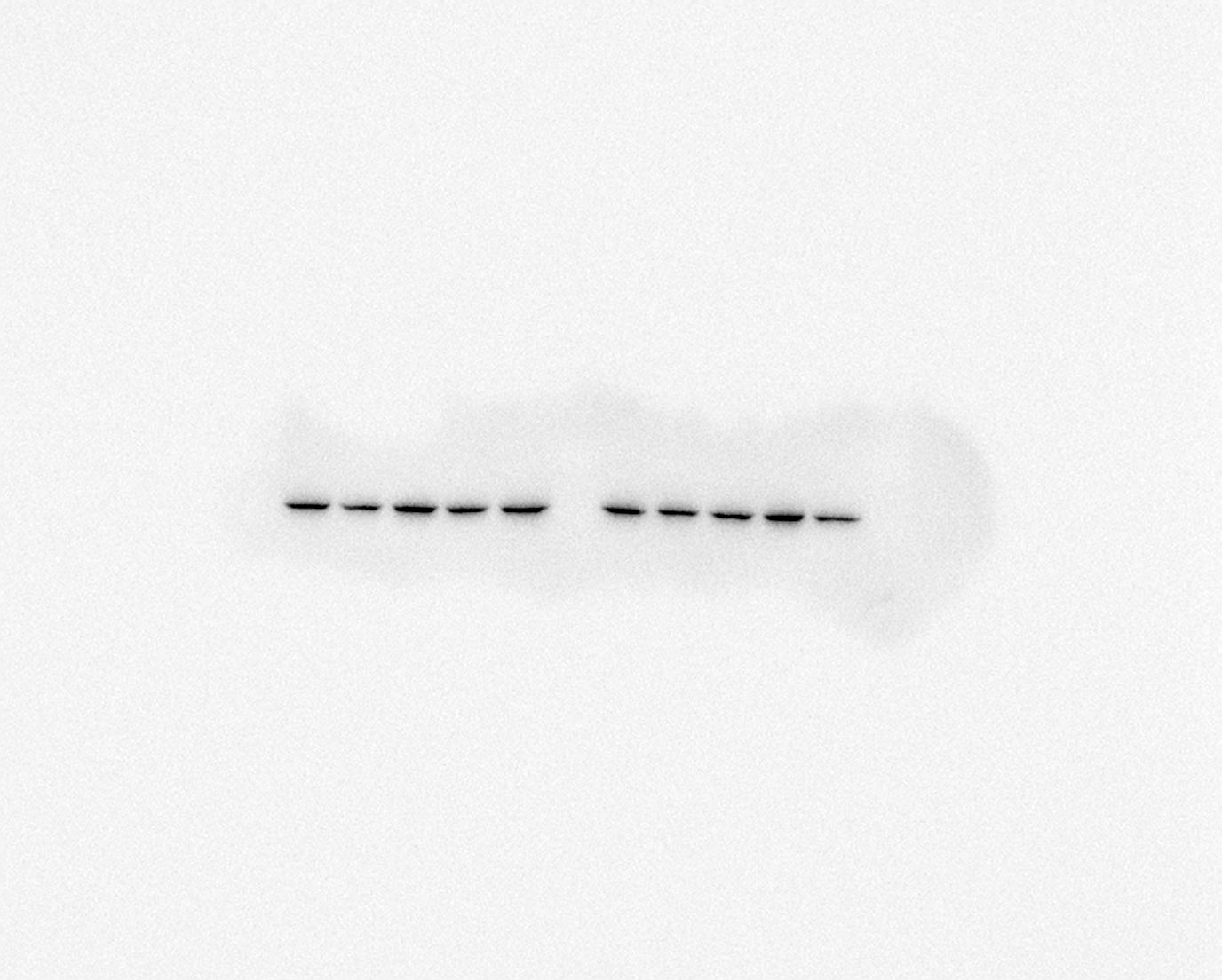

Supplement: Figure 4—source data 1. [file elife-86075-fig4-data1.zip › Figure 4-source data/C/raw unedited gels or blots/Tubulin.tif]

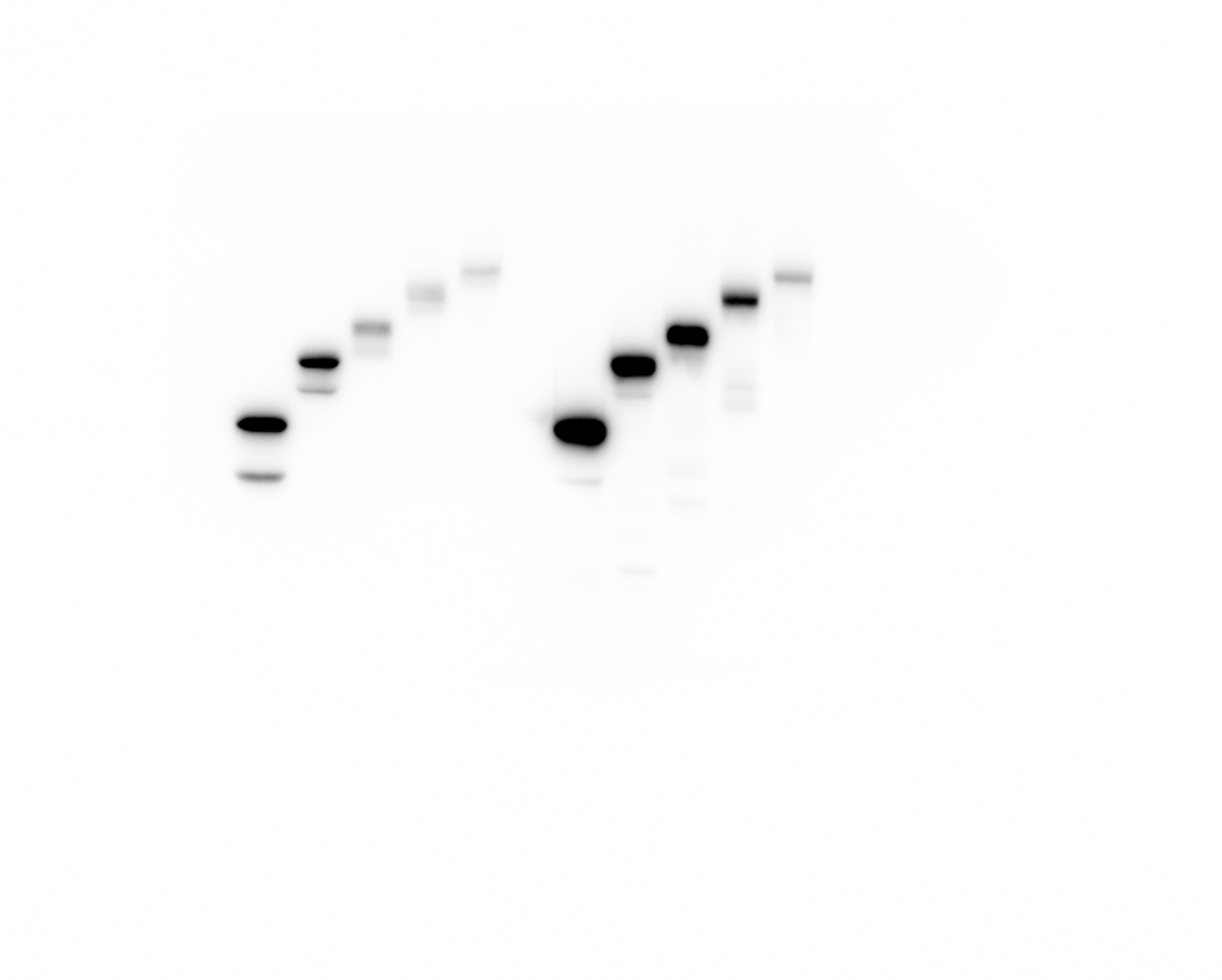

Supplement: Figure 4—source data 1. [file elife-86075-fig4-data1.zip › Figure 4-source data/C/raw unedited gels or blots/TAP.tif]

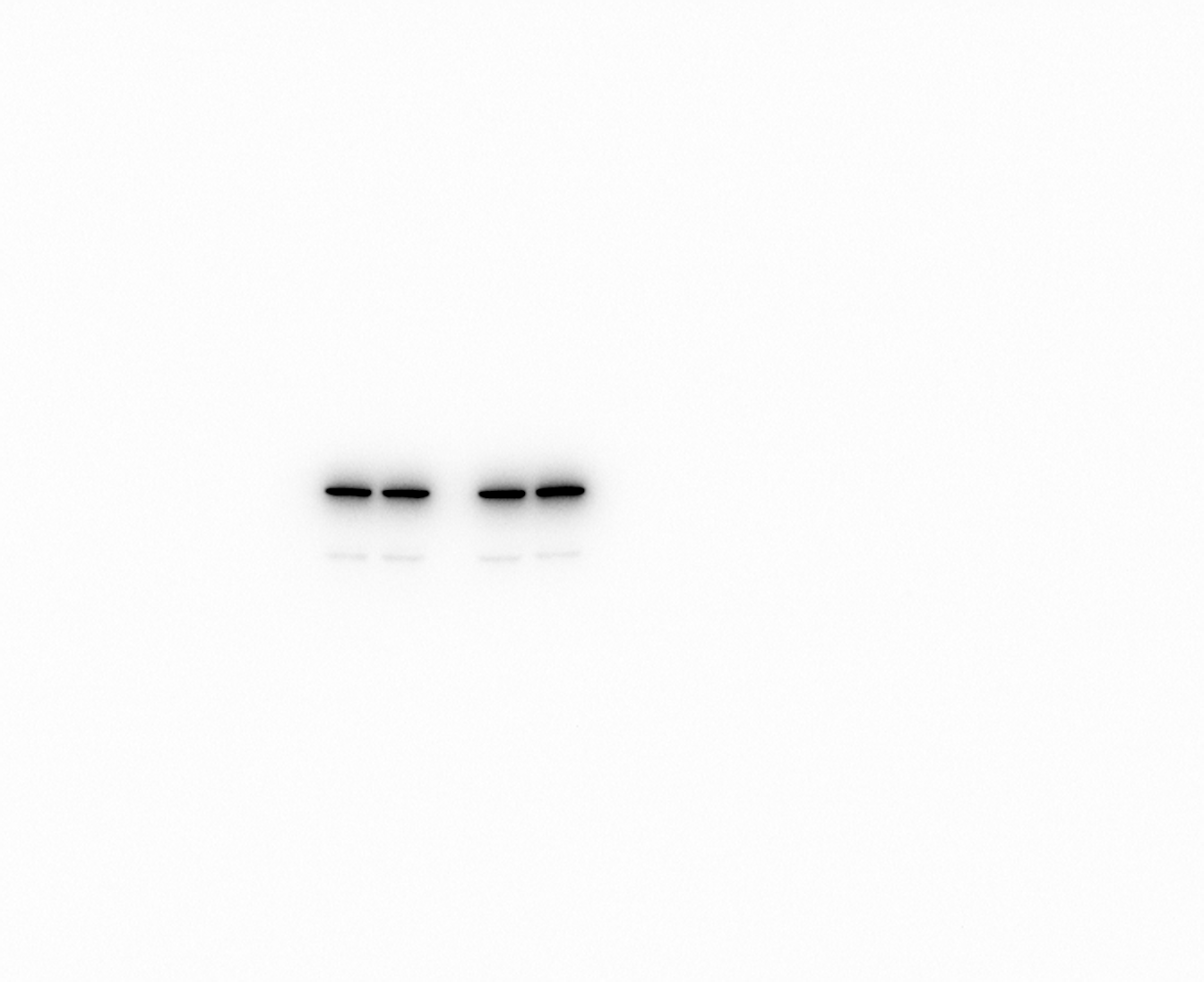

Supplement: Figure 4—source data 1. [file elife-86075-fig4-data1.zip › Figure 4-source data/E/raw unedited gels or blots/Tubulin.Tif]

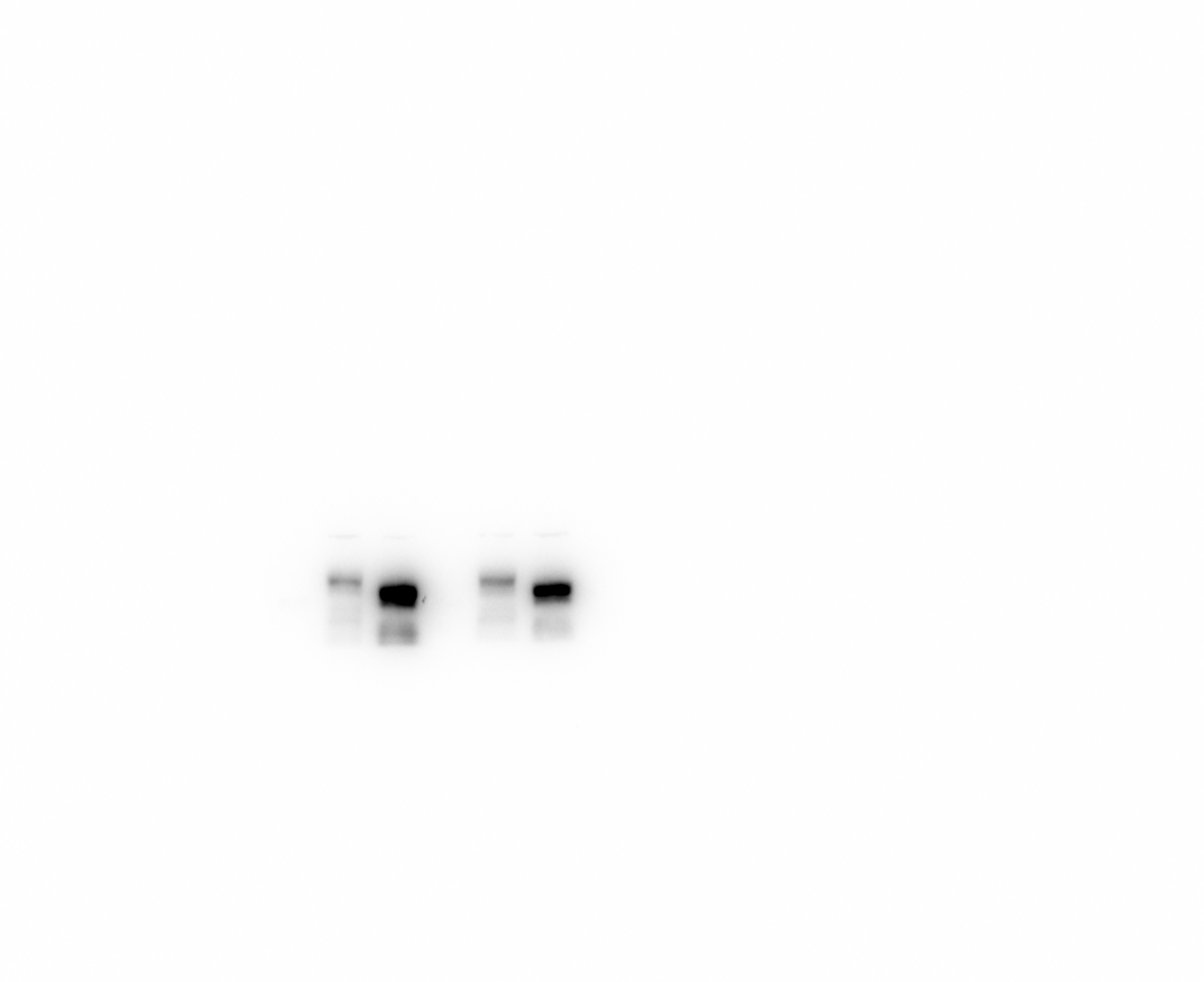

Supplement: Figure 4—source data 1. [file elife-86075-fig4-data1.zip › Figure 4-source data/E/raw unedited gels or blots/Myc.Tif]

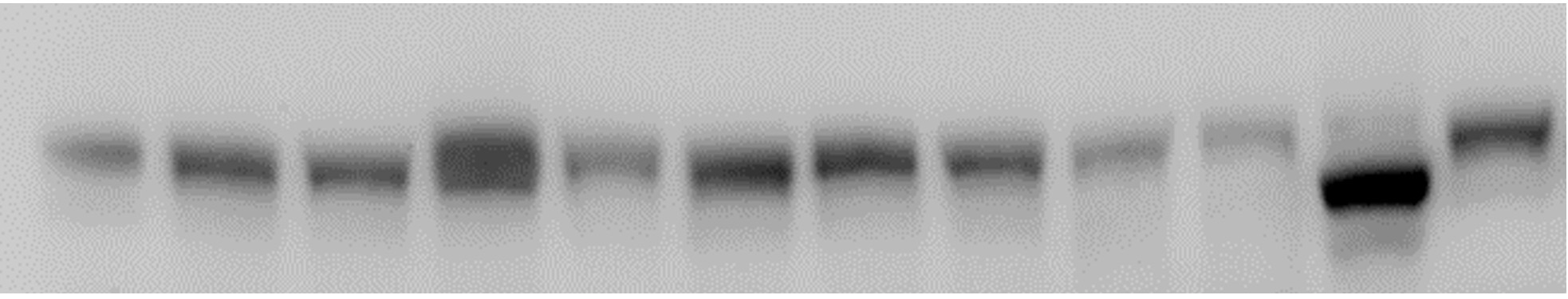

Supplement: Figure 4—source data 1. [file elife-86075-fig4-data1.zip › Figure 4-source data/B/raw unedited gels or blots/Myc.jpg]

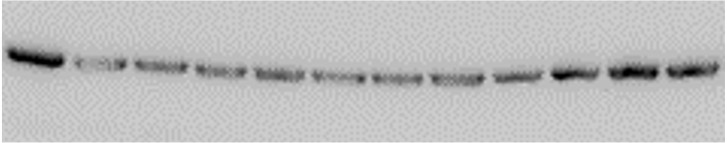

Supplement: Figure 4—source data 1. [file elife-86075-fig4-data1.zip › Figure 4-source data/B/raw unedited gels or blots/Tubulin.jpg]

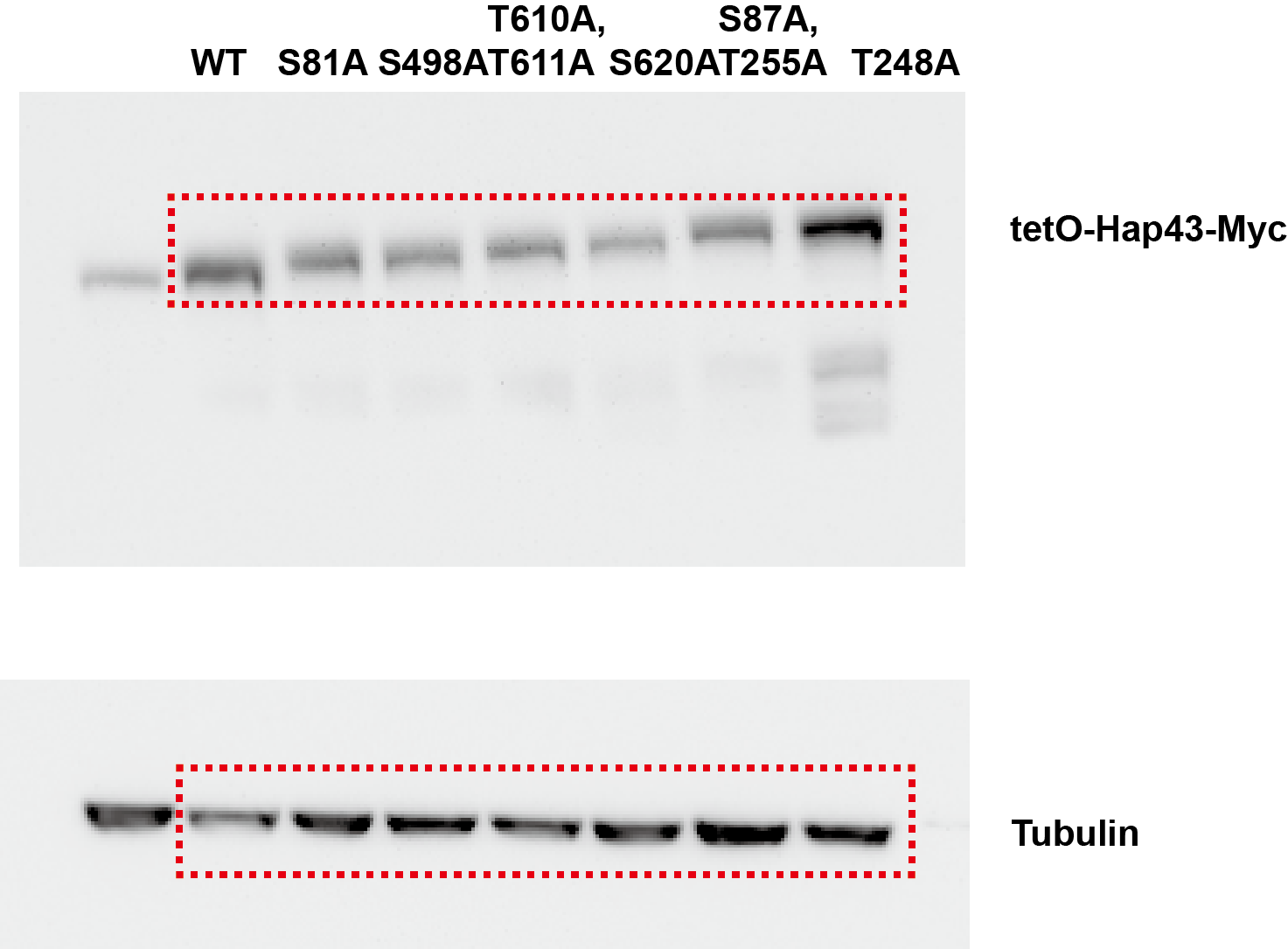

Supplement: Figure 4—figure supplement 1—source data 1. [file elife-86075-fig4-figsupp1-data1.zip › Figure 4ΓÇöfigure supplement 1-source data/A/Figure 4ΓÇöfigure supplement 1A with uncropped gels or blots.tif]

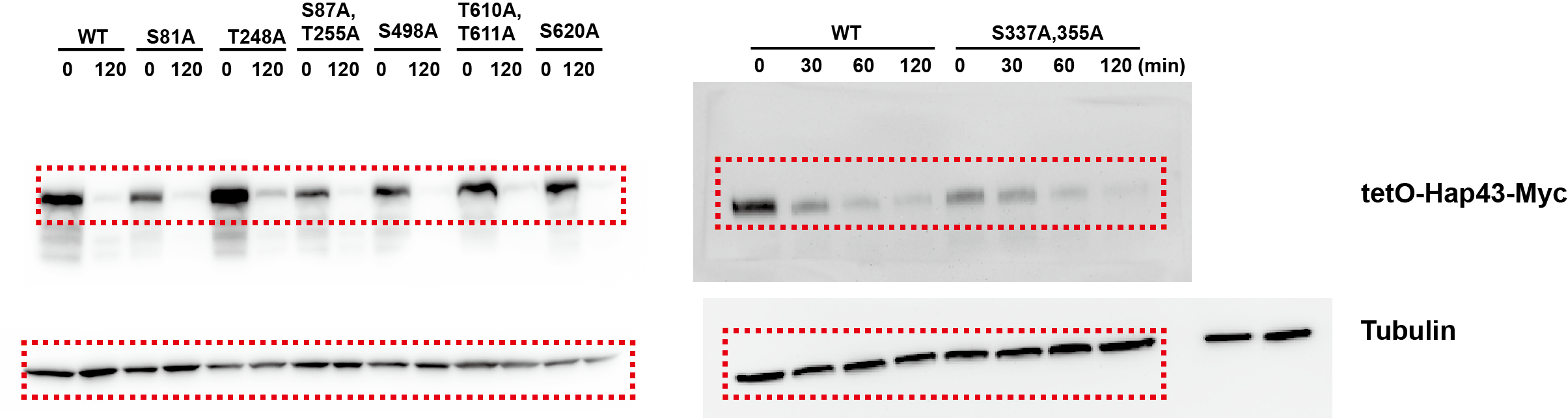

Supplement: Figure 4—figure supplement 1—source data 1. [file elife-86075-fig4-figsupp1-data1.zip › Figure 4ΓÇöfigure supplement 1-source data/B/Figure 4ΓÇöfigure supplement 1B with uncropped gels or blots.tif]

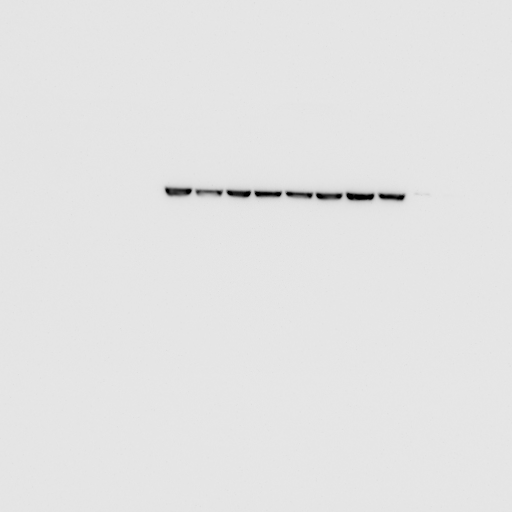

Supplement: Figure 4—figure supplement 1—source data 1. [file elife-86075-fig4-figsupp1-data1.zip › Figure 4ΓÇöfigure supplement 1-source data/A/raw unedited gels or blots/Tubulin.tif]

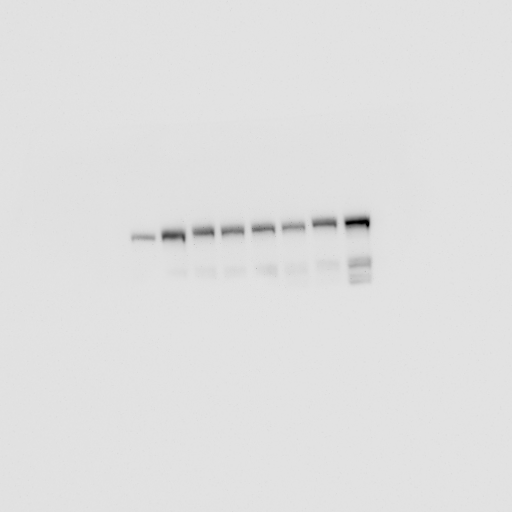

Supplement: Figure 4—figure supplement 1—source data 1. [file elife-86075-fig4-figsupp1-data1.zip › Figure 4ΓÇöfigure supplement 1-source data/A/raw unedited gels or blots/Myc.tif]

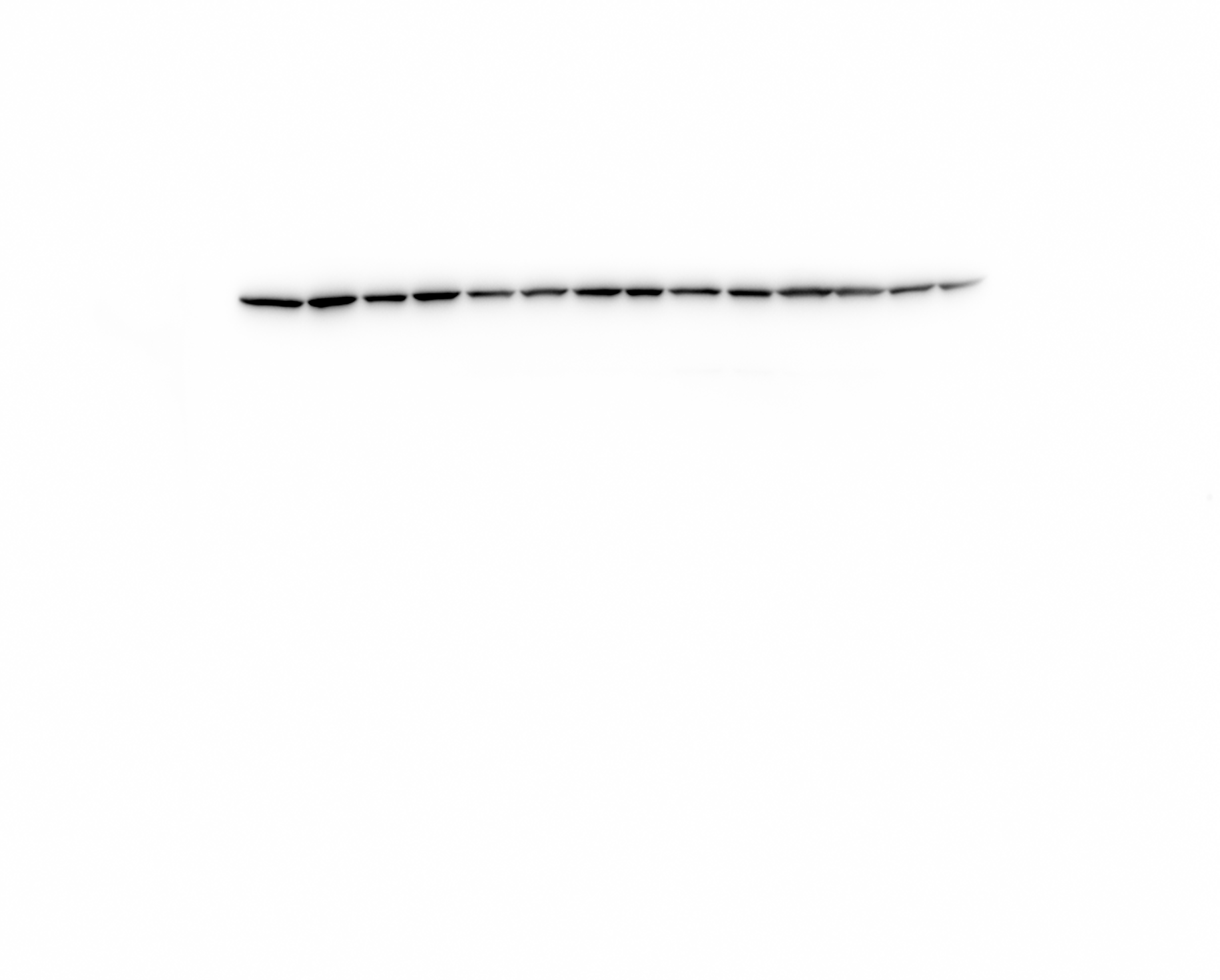

Supplement: Figure 4—figure supplement 1—source data 1. [file elife-86075-fig4-figsupp1-data1.zip › Figure 4ΓÇöfigure supplement 1-source data/B/raw unedited gels or blots/Tubulin_left panel..tif]

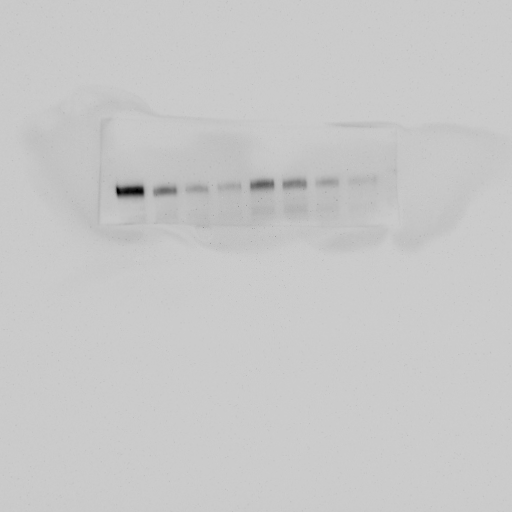

Supplement: Figure 4—figure supplement 1—source data 1. [file elife-86075-fig4-figsupp1-data1.zip › Figure 4ΓÇöfigure supplement 1-source data/B/raw unedited gels or blots/Myc_right panel.tif]

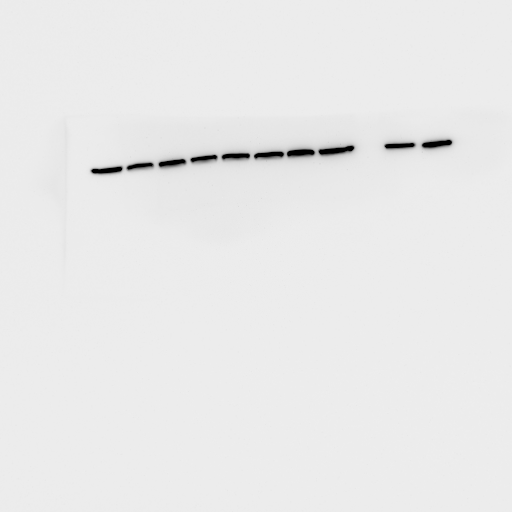

Supplement: Figure 4—figure supplement 1—source data 1. [file elife-86075-fig4-figsupp1-data1.zip › Figure 4ΓÇöfigure supplement 1-source data/B/raw unedited gels or blots/Tubulin_right panel..tif]

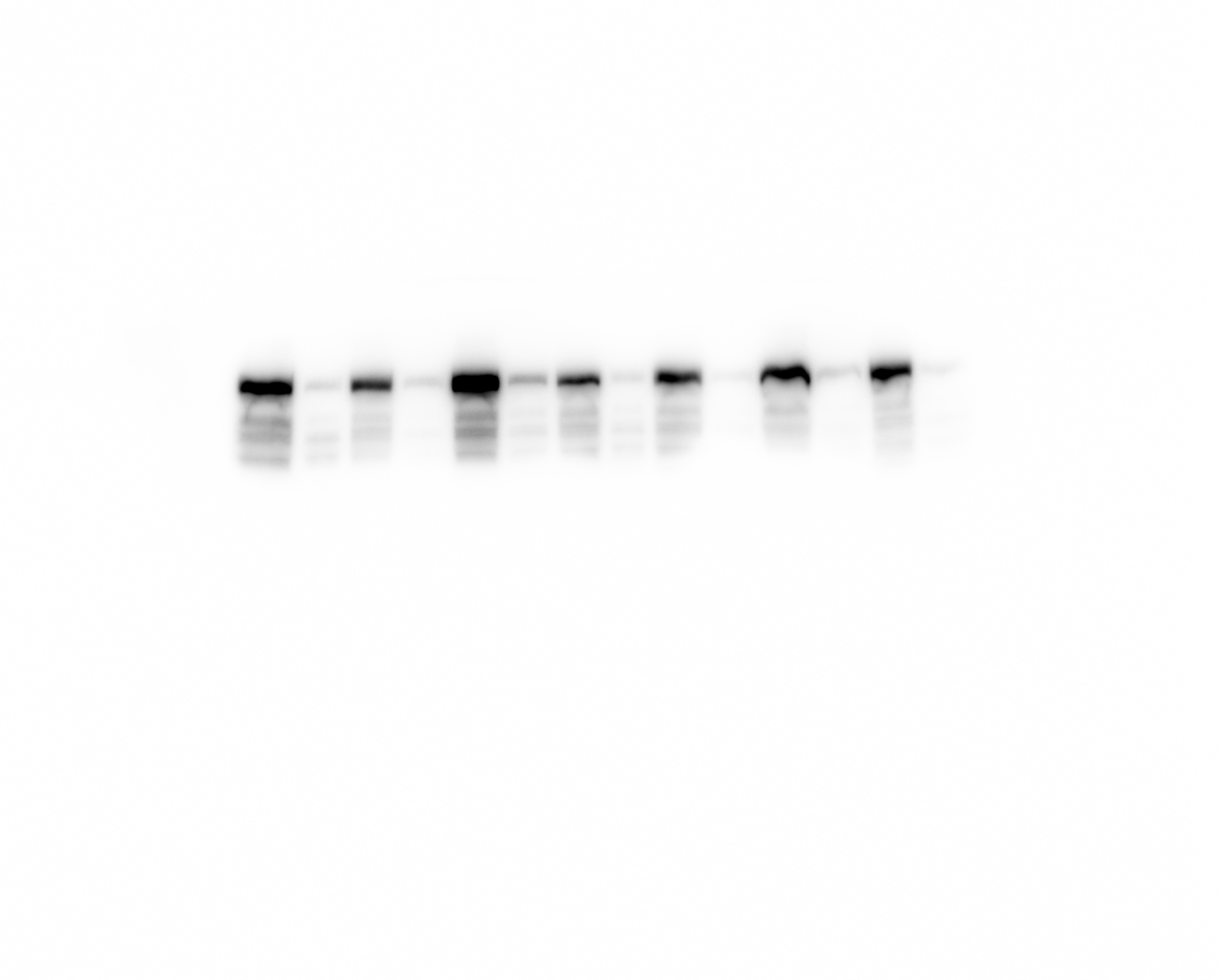

Supplement: Figure 4—figure supplement 1—source data 1. [file elife-86075-fig4-figsupp1-data1.zip › Figure 4ΓÇöfigure supplement 1-source data/B/raw unedited gels or blots/Myc_left panel..tif]

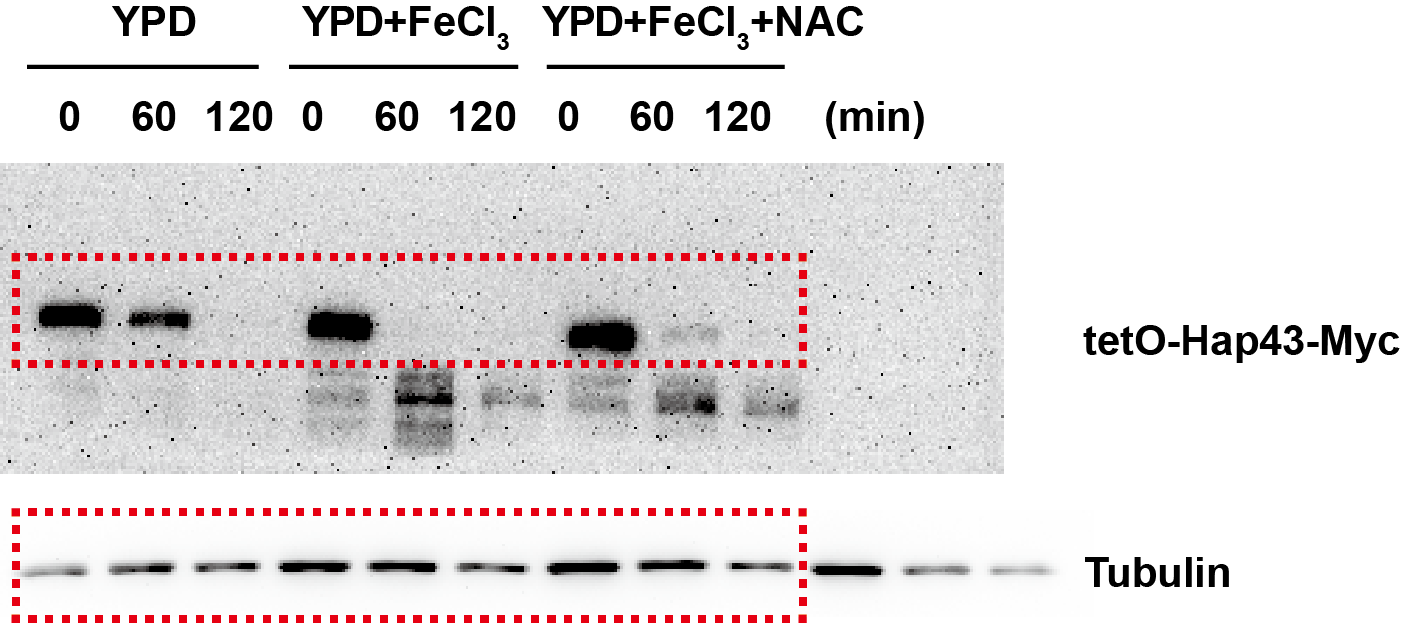

Supplement: Figure 5—source data 1. [file elife-86075-fig5-data1.zip › Figure 5-source data/C/Figure 5C with uncropped gels or blots.tif]

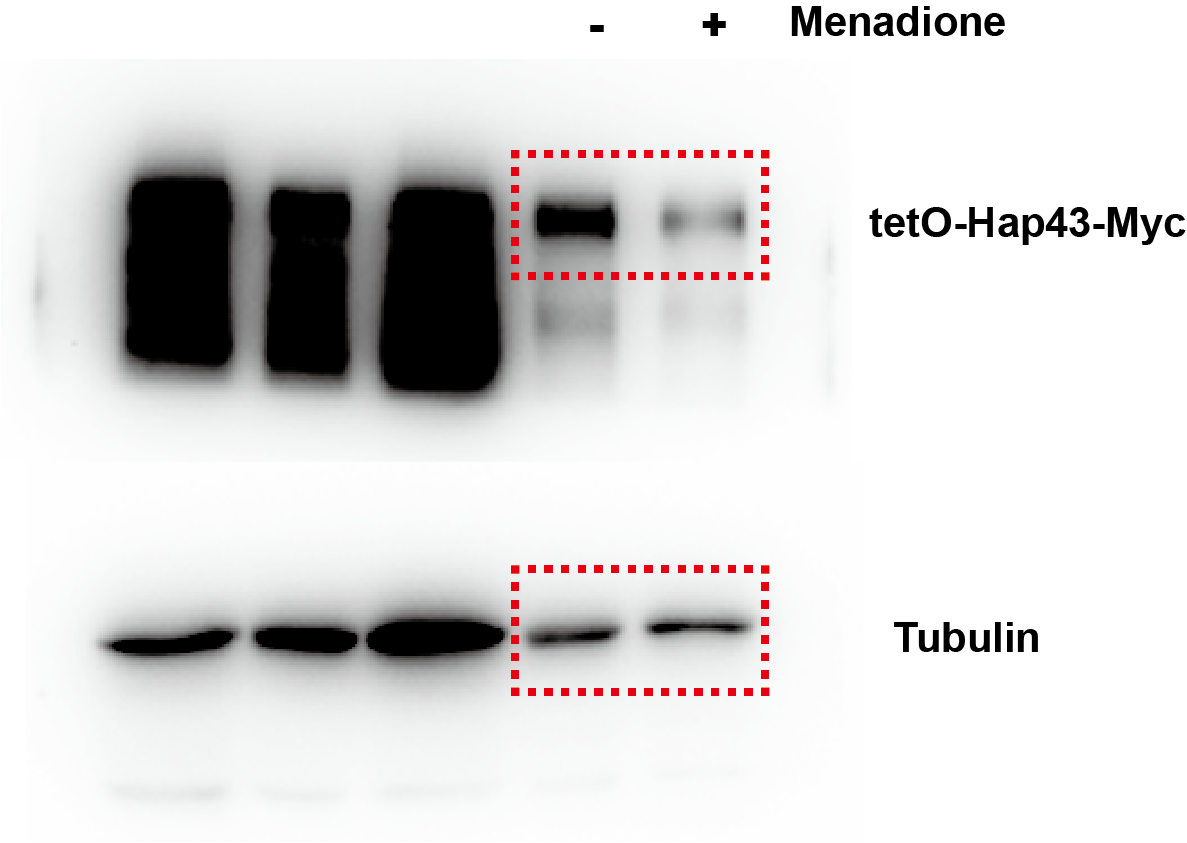

Supplement: Figure 5—source data 1. [file elife-86075-fig5-data1.zip › Figure 5-source data/D/Figure 5D with uncropped gels or blots.tif]

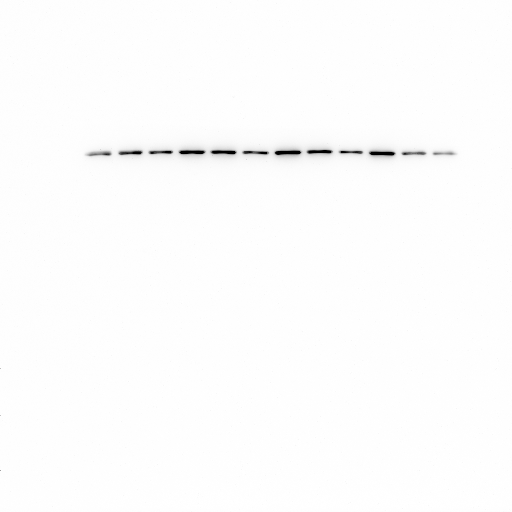

Supplement: Figure 5—source data 1. [file elife-86075-fig5-data1.zip › Figure 5-source data/C/raw unedited gels or blots/Tubulin.tif]

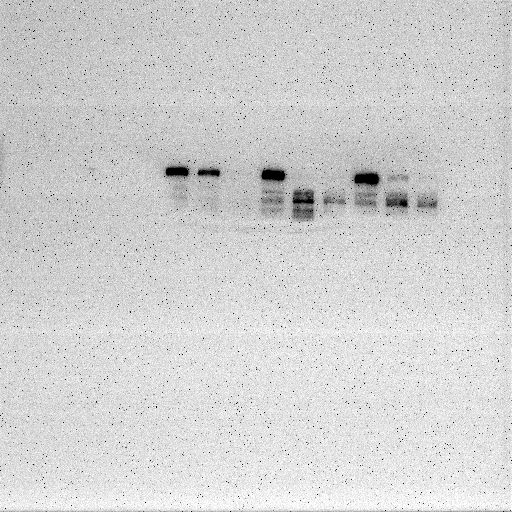

Supplement: Figure 5—source data 1. [file elife-86075-fig5-data1.zip › Figure 5-source data/C/raw unedited gels or blots/Myc.tif]

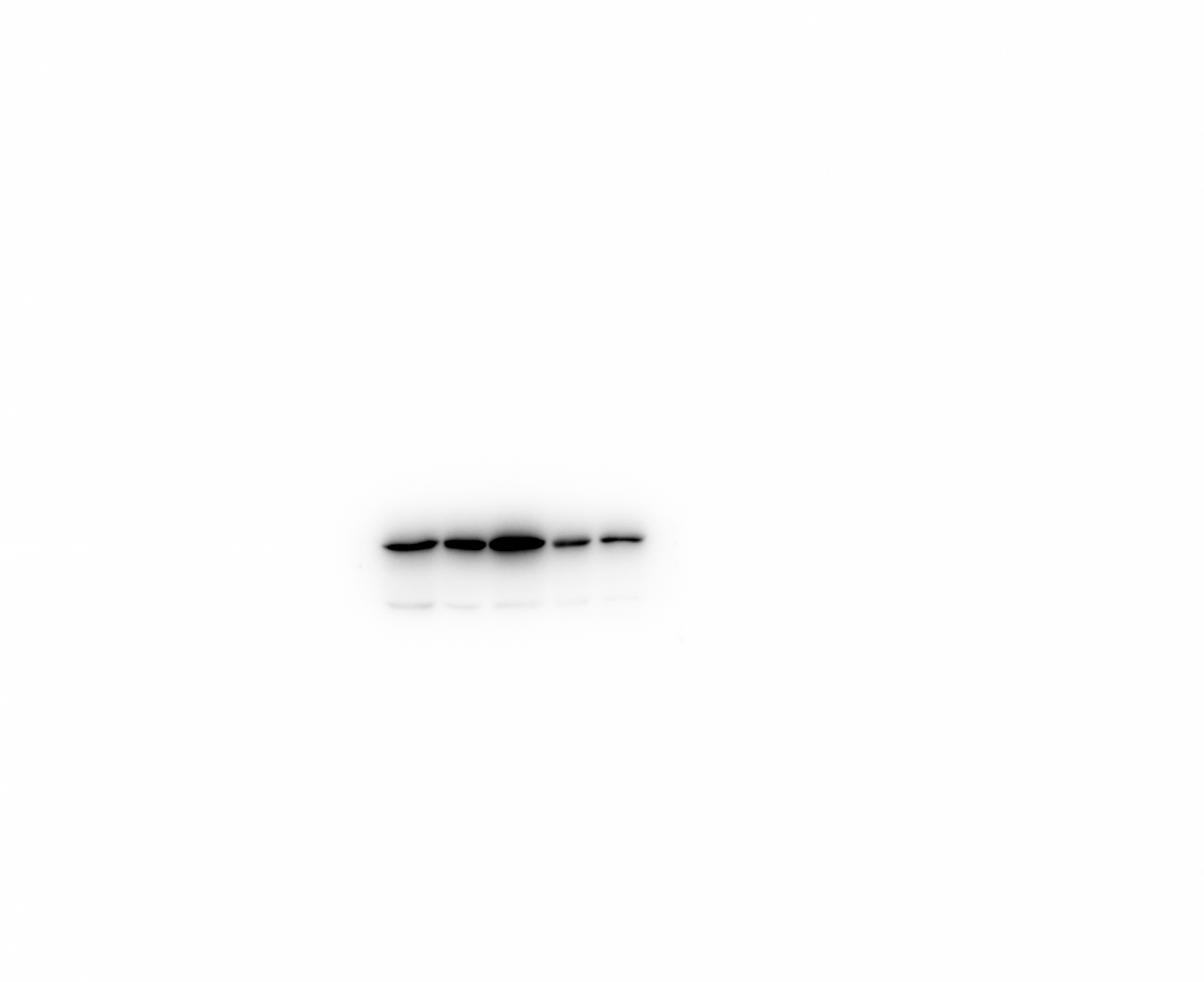

Supplement: Figure 5—source data 1. [file elife-86075-fig5-data1.zip › Figure 5-source data/D/raw unedited gels or blots/Tubulin.Tif]

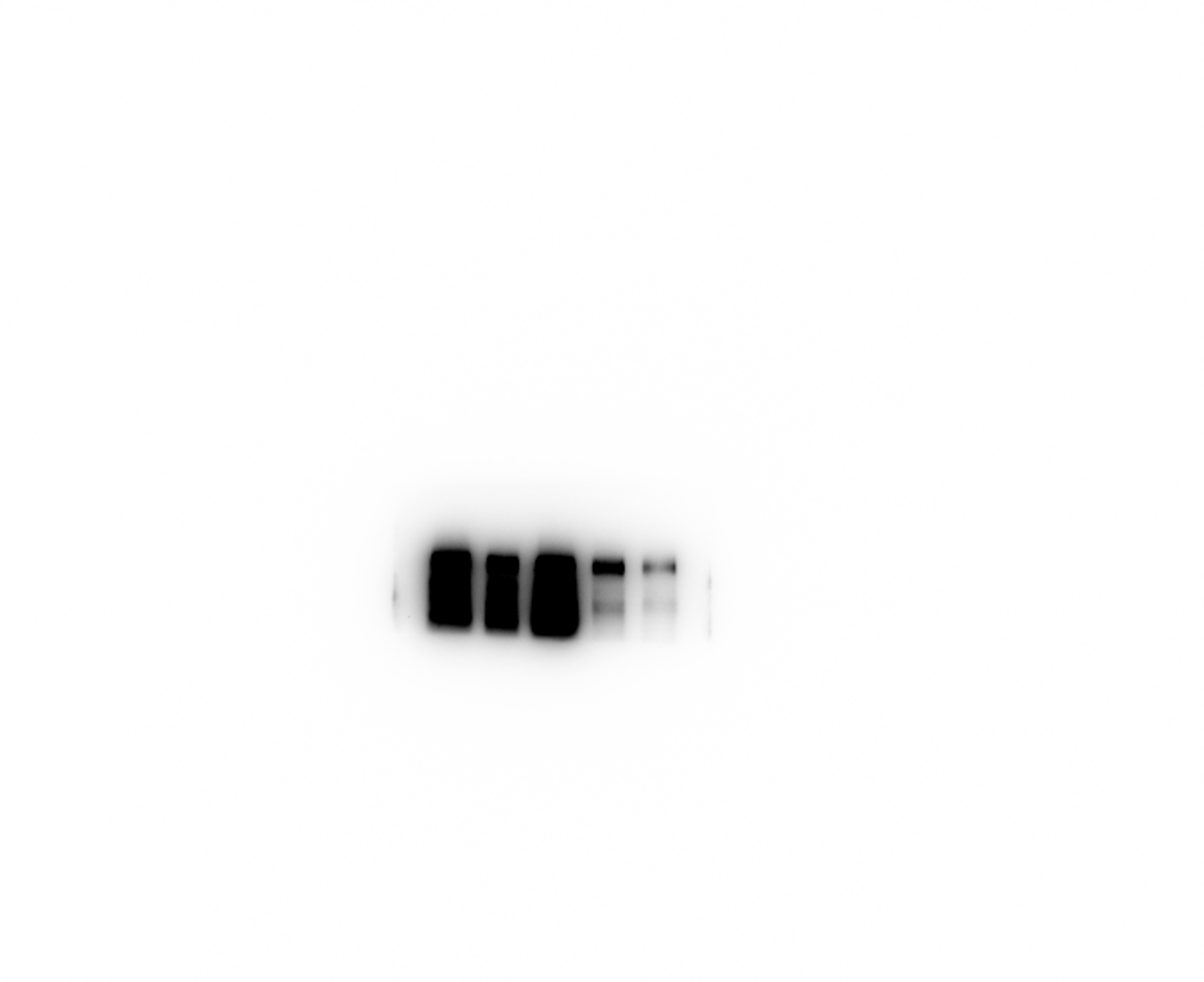

Supplement: Figure 5—source data 1. [file elife-86075-fig5-data1.zip › Figure 5-source data/D/raw unedited gels or blots/Myc.Tif]
