# Supplementary material for: Homeostatic control of an iron repressor in a GI tract resident
Source: eLife. 2023 May 25;12:e86075. doi: 10.7554/eLife.86075 (PMC10259491; doi:10.7554/eLife.86075)
Supplement: Supplementary file 1. [file elife-86075-supp1.docx]

**a. Strains used in this study.**

| **Strain** | **Relevant Genotype** | **Full Genotype** | **Reference** |
| --- | --- | --- | --- |
| SC5314 | *Wild type* |  | Noble et al., 2010 |
| SN250 | *Wild type* | *leu2Δ::C.m.LEU2/leu2Δ::C.d.HIS1, his1Δ/his1Δ, arg4Δ/arg4Δ, leu2D/leu2Δ ura3Δ/URA3, iro1Δ/IRO1* | Noble et al., 2010 |
| SN694 | *hap43∆/∆* | *hap43∆::C.m.LEU2/ hap43∆::C.d.HIS1, his1Δ/his1Δ, arg4Δ/arg4Δ, leu2Δ/leu2Δ, ura3Δ/URA3, iro1Δ/IRO1* | Noble et al., 2010 |
| SN856 | Hap43-Myc*/HAP43* | *HAP43-13xMyc/HAP43, leu2Δ::C.m.LEU2/leu2Δ::C.d.HIS1, his1Δ/his1Δ, arg4Δ/arg4Δ, leu2D/leu2Δ ura3Δ/URA3, iro1Δ/IRO1* | Noble et al., 2010 |
| SN913 | *ssn3∆/∆* | *ssn3Δ::C.m.LEU2/ssn3Δ::C.d.HIS1, his1Δ/his1Δ, arg4Δ/arg4Δ, leu2Δ/leu2Δ, ura3Δ/URA3, iro1Δ/IRO1* | Noble et al., 2010 |
| CB37 | Hap43-Myc*/HAP43, ssn3∆/∆* | *HAP43-13xMyc/HAP43, his1Δ/his1Δ, ssn3 Δ::C.m.LEU2/ssn3Δ::C.d.HIS1, arg4Δ/arg4Δ, leu2Δ/leu2Δ, ura3Δ/URA3, iro1Δ/IRO* | this study |
| CB44 | Hap43-Myc*/HAP43,* *ssn3^D325A^* | *HAP43-13xMyc/HAP43, his1Δ/his1Δ, ssn3 Δ::C.m.LEU2/ssn3Δ::C.d.HIS1, arg4Δ/arg4Δ, leu2Δ/leu2Δ: ssn3^D325A^-C.d.ARG4, ura3Δ/URA3, iro1Δ/IRO* | this study |
| CB54 | Hap43-TAP*/HAP43* | *HAP43-TAP/HAP43, leu2Δ::C.m.LEU2/leu2Δ::C.d.HIS1, his1Δ/his1Δ, arg4Δ/arg4Δ, leu2D/leu2Δ ura3Δ/URA3, iro1Δ/IRO1* | this study |
| CB247 | tetO-Hap43-Myc*/ hap43∆* | *tetO-HAP43-13xMyc/ADH1, his1Δ/his1Δ, hap43Δ::C.m.LEU2/hap43Δ::C.d.HIS1, arg4Δ/arg4Δ, leu2Δ/leu2**Δ, ura3Δ/URA3, iro1Δ/IRO* | this study |
| CB298 | tetO-Hap43-Myc*/HAP43, ssn3∆/∆* | *tetO-HAP43-13xMyc/ADH1, his1Δ/his1Δ, ssn3Δ::C.m.LEU2/ssn3Δ::C.d.HIS1, arg4Δ/arg4Δ, leu2Δ/leu2Δ, ura3Δ/URA3, iro1Δ/IRO* | this study |
| CB329 | Hap43-Myc*/HAP43,* Ssn3-TAP*/SSN3* | *HAP43-13xMyc/HAP43, SSN3-TAP/SSN3, his1Δ/his1Δ, arg4Δ/arg4Δ, leu2Δ/leu2Δ, ura3Δ/URA3, iro1Δ/IRO* | this study |
| CB432  (*Δ230*) | Hap43(1-230aa)-TAP*/HAP43* | *HAP43(1-230aa)-TAP/HAP43, leu2Δ::C.m.LEU2/leu2Δ::C.d.HIS1, his1Δ/his1Δ, arg4Δ/arg4Δ, leu2D/leu2Δ ura3Δ/URA3, iro1Δ/IRO1* | this study |
| CB444 | tetO-Hap43-Myc*, erg6∆* | *tetO-HAP43-13xMyc/ADH1, his1Δ/his1Δ, erg6Δ::C.m.LEU2/ERG6, arg4Δ/arg4Δ, leu2Δ/leu2Δ, ura3Δ/URA3, iro1Δ/IRO* | this study |
| CB453 | Hap43-Myc*/HAP43,* tetO-HA-Ub | *HAP43-13xMyc/HAP43, tetO-HA-Ub/ADH1, his1Δ/his1Δ, arg4Δ/arg4Δ, leu2Δ/leu2Δ, ura3Δ/URA3, iro1Δ/IRO* | this study |
| CB494 | Hap43-Myc*/HAP43,* tetO-HA-Ub, *ssn3**∆/∆* | *HAP43-13xMyc/HAP43, tetO-HA-Ub/ADH1, his1Δ/his1Δ, ssn3 Δ::C.m.LEU2/ssn3Δ::C.d.HIS1, arg4Δ/arg4Δ, leu2Δ/leu2Δ, ura3Δ/URA3, iro1Δ/IRO* | this study |
| CB432  (*Δ330*) | Hap43(1-330aa)-TAP*/HAP43* | *HAP43(1-330aa)-TAP/HAP43, leu2Δ::C.m.LEU2/leu2Δ::C.d.HIS1, his1Δ/his1Δ, arg4Δ/arg4Δ, leu2D/leu2Δ ura3Δ/URA3, iro1Δ/IRO1* | this study |
| CB432  (*Δ400*) | Hap43(1-400aa)-TAP*/HAP43* | *HAP43(1-400aa)-TAP/HAP43, leu2Δ::C.m.LEU2/leu2Δ::C.d.HIS1, his1Δ/his1Δ, arg4Δ/arg4Δ, leu2D/leu2Δ ura3Δ/URA3, iro1Δ/IRO1* | this study |
| CB432  (*Δ504*) | Hap43(1-504aa)-TAP*/HAP43* | *HAP43(1-504aa)-TAP/HAP43, leu2Δ::C.m.LEU2/leu2Δ::C.d.HIS1, his1Δ/his1Δ, arg4Δ/arg4Δ, leu2D/leu2Δ ura3Δ/URA3, iro1Δ/IRO1* | this study |
| CB534 | tetO-Hap43(S337A,355A)-Myc*/ hap43∆* | *tetO-HAP43(S81A)-13xMyc/ADH1, his1Δ/his1Δ, hap43Δ::C.m.LEU2/hap43Δ::C.d.HIS1, arg4Δ/arg4Δ, leu2Δ/leu2Δ, ura3Δ/URA3, iro1Δ/IRO* | this study |
| CB561 | tetO-Hap43(S81A)-Myc*/ hap43∆* | *tetO-HAP43(S81A)-13xMyc/ADH1, his1Δ/his1Δ, hap43Δ::C.m.LEU2/hap43Δ::C.d.HIS1, arg4Δ/arg4Δ, leu2Δ/leu2Δ, ura3Δ/URA3, iro1Δ/IRO* | this study |
| CB562 | tetO-Hap43(S248A)-Myc*/ hap43∆* | *tetO-HAP43(S248A)-13xMyc/ADH1, his1Δ/his1Δ, hap43Δ::C.m.LEU2/hap43Δ::C.d.HIS1, arg4Δ/arg4Δ, leu2Δ/leu2Δ, ura3Δ/URA3, iro1Δ/IRO* | this study |
| CB563 | tetO-Hap43(S498A)-Myc*/ hap43∆* | *tetO-HAP43(S498A)-13xMyc/ADH1, his1Δ/his1Δ, hap43Δ::C.m.LEU2/hap43Δ::C.d.HIS1, arg4Δ/arg4Δ, leu2Δ/leu2Δ, ura3Δ/URA3, iro1Δ/IRO* | this study |
| CB564 | tetO-Hap43(T610A,T611A)-Myc*/ hap43∆* | *tetO-HAP43(S81A)-13xMyc/ADH1, his1Δ/his1Δ, hap43Δ::C.m.LEU2/hap43Δ::C.d.HIS1, arg4Δ/arg4Δ, leu2Δ/leu2Δ, ura3Δ/URA3, iro1Δ/IRO* | this study |
| CB565 | tetO-Hap43(S620A)-Myc*/ hap43∆* | *tetO-HAP43(S81A)-13xMyc/ADH1, his1Δ/his1Δ, hap43Δ::C.m.LEU2/hap43Δ::C.d.HIS1, arg4Δ/arg4Δ, leu2Δ/leu2Δ, ura3Δ/URA3, iro1Δ/IRO* | this study |
| CB602 | tetO-Hap43(S87A,T255A)-Myc*/ hap43∆* | *tetO-HAP43(S81A)-13xMyc/ADH1, his1Δ/his1Δ, hap43Δ::C.m.LEU2/hap43Δ::C.d.HIS1, arg4Δ/arg4Δ, leu2Δ/leu2Δ, ura3Δ/URA3, iro1Δ/IRO* | this study |
| CB709 | *HAP43/hap43∆* | *arg4Δ/arg4Δ, leu2Δ/leu2Δ::**HAP43-13xMyc-C.d.ARG4,* *his1Δ/his1Δ, ura3Δ/URA3, iro1Δ/IRO1, hap43 Δ::C.d.HIS1/hap43Δ::C.m.LEU2* | this study |
| CB710 | *HAP43m29/hap43∆* | *arg4Δ/arg4Δ, leu2Δ/leu2Δ::HAP43m29-13xMyc-C.d.ARG4, his1Δ/his1Δ, ura3Δ/URA3, iro1Δ/IRO1, hap43Δ::C.d.HIS1/hap43Δ::C.m.LEU2* | this study |
| CB776 | tetO-Hap43tr-Myc*/ hap43∆* | *HAP43tr-13xMyc/HAP43, leu2Δ::C.m.LEU2/leu2Δ::C.d.HIS1, his1Δ/his1Δ, arg4Δ/arg4Δ, leu2D/leu2Δ ura3Δ/URA3, iro1Δ/IRO1* | this study |
| CB856 | *HAP43/hap43∆* | *ACT1/pACT1-HAP43-13xMyc-NAT1-tACT1,*  *his1Δ/his1Δ, ura3Δ/URA3, iro1Δ/IRO1, hap43 Δ::C.d.HIS1/hap43Δ::C.m.LEU2* | this study |
| CB857 | *HAP43tr/hap43∆* | *ACT1/pACT1-HAP43tr-13xMyc-NAT1-tACT1,his1Δ/his1Δ, ura3Δ/URA3, iro1Δ/IRO1, hap43 Δ::C.d.HIS1/hap43Δ::C.m.LEU2* | this study |
| CB1113 | *HAP43m4/hap43∆* | *ACT1/**pACT1-HAP43m4-13xMyc-NAT1-tACT1, his1Δ/his1Δ, ura3Δ/URA3, iro1Δ/IRO1, hap43 Δ::C.d.HIS1/hap43Δ::C.m.LEU2* | this study |

**b. Primers used in this study.**

| **Primer** | **Purpose** | **Sequence (5’-3’)** |
| --- | --- | --- |
| CBO158 | qPCR for *ACT1*, forward | GTGGTACTACCATGTTCCCAGG |
| CBO159 | qPCR *for ACT1*, reverse | GATAGAACCACCAATCCAGACAGAG |
| CBO500 | Common primer for *Leu2* 5' flank, forward | GGCGAATTGGAGCTCCACCGCGGTGGCGGCCGCTCTAGAACTAGTGGATCGTTTAAACTTGGTAGATTTACAACTGAAGCCG |
| CBO501 | Common primer for *Leu2* 3' flank, reverse | GTCGACGGTATCGATAAGCTTGATATCGAATTCCTGCAGCCCGGGGGATCGTTTAAACTCGAAAACGATGTTTGCACCACCG |
| CBO503 | Common primer for FRT-FLP-SAT1-FRT cassette, forward | GCAGATATCCATCACACTGGCTTGGGTACCGGGCCCCCCCTCGAGGA |
| CBO504 | Common primer for CdARG4, reverse | GCAGTCGTTGTCGACGAGGAGCAACAGTAATGAAAGCAGTCAAAGGGCTC |
| CBO614 | Common 5' verification primer for Myc-tagging, reverse | GCCACCTAATGTGGGTTACACT |
| CBO615 | Common 3' verification primer for Myc-tagging, forward | CTCAGTGCAGAAACAGGAAAAGAG |
| CBO838 | Hap43-13x Myc 5' flank, forward | GGCGAATTGGAGCTCCACCGCGGTGGCGGCCGCTCTAGAACTAGTGGATCGTTTAAACCGTCAACAAGTCAACTAACAGATG |
| CBO839 | Hap43-13x Myc 5' flank, reverse | GTTCACCGTTAATTAACCCGGGGATCCGACATATGTTGTTGTGTTGCTGTTGTTGTTGT |
| CBO2454 | qPCR for *HAP43*, forward | CTCAAAATAGAGCTGCACAGAGAG |
| CBO2455 | qPCR *for HAP43*, reverse | GTTTTCTTCCAGGTTTTGGTCTTG |
| CBO2563 | qPCR for *CAT1*, forward | TTGGTCAACACGGTCCATTG |
| CBO2564 | qPCR *for CAT1*, reverse | GCGTGGACAACTCTTTCTGG |
| CBO2565 | qPCR for *SOD2*, forward | CGCTGCTTCCAAGACTTTCA |
| CBO2566 | qPCR *for SOD2*, reverse | ATTTGGCTTCAACGGCTTGT |
| CBO2569 | qPCR for *GSH1*, forward | ATACCAACCACGGCACATTG |
| CBO2570 | qPCR *for GSH1*, reverse | ACTCAGTAATGTCAGCACCCA |
| CBO2571 | qPCR for *TTR1*, forward | ACCAAACCAGTTTTCATTGCC |
| CBO2572 | qPCR *for TTR1*, reverse | ACCGTCGTCAACTTCGTCTA |
| CBO2751 | *HAP43* reverse complement, forward | ATTTTATGATGGAATGAATGGGATGAATCATCAAACAAGAGAAAATACCCG |
| CBO2754 | *HAP43* reverse complement, reverse | GCTGACACAAACGGGAACAGAAATAGTTTCAAGTCGTGCAGAATGTTAC |
| CBO2755 | Backbone for *HAP43* addback, forward | CTATTTCTGTTCCCGTTTGTGTCAGCACATTTCTGTACCGCAAATGTATCG |
| CBO2756 | Backbone for *HAP43* addback, reverse | CATCCCATTCATTCCATCATAAAATGAAAAGGGGAGTATTTCTGGAGTG |
| CBO2757 | *HAP43-*mutation 29 ORF-13x Myc, reverse | ATTATAAGCTCTTCTATCCAATTCTCTCAAAACATTAGCAACAGCTTG |
| CBO2758 | *HAP43-*mutation 29 ORF-13x Myc, forward | GAATTGGATAGAAGAGCTTATAATCGGATCCCCGGGTTAATTAACGGT |
| CBO2863 | qPCR for *CAT1* promoter, forward | AAACCAATCACTCTTTCATTAATAAGC |
| CBO2864 | qPCR for *CAT1* promoter, reverse | TTTTTAGCATTACCCCAGTTCATA |
| CBO2870 | qPCR for *TRR1* promoter, forward | TTACAAAAGAAGATGGCCAATACA |
| CBO2871 | qPCR for *TRR1* promoter, reverse | GCACGCTAATCAAAAGAAATACAA |
| CBO2874 | qPCR for *SOD2* promoter, forward | ACATATTGTCTCCCAATGTTACGA |
| CBO2875 | qPCR for *SOD2* promoter, reverse | ACTGGAGTCAAACCTAACAAGGTC |
| CBO2878 | qPCR for *GSH1* promoter, forward | TAACACATGAATATTGGTCAATTGGAA |
| CBO2879 | qPCR for *GSH1* promoter, reverse | GTCTTGAAAAGATTTGGTTACTAGC |
| CBO2888 | qPCR for *HAP43-mut*, forward | TGCTCCACCAGTTTCTGTTAATAC |
| CBO2889 | qPCR *for HAP43-mut*, reverse | GTTTTCTACCTGGTTTTGGTCTTG |
| CBO3023 | qPCR for *DUOX2*, forward | AGCTATGCCCTCATCCAATTAC |
| CBO3024 | qPCR *for DUOX2*, reverse | CACACTGATCTCCACCTTCTTC |
| CBO3107 | qPCR for *GAPDH*, forward | AGGTCGGTGTGAACGGATTTG |
| CBO3108 | qPCR *for GAPDH*, reverse | TGTAGACCATGTAGTTGAGGTCA |
| CBO3373  CBO3374  CBO3383  CBO3384 | qPCR for *tetO-HAP43*, forward  qPCR for *tetO-HAP43*, reverse  Specific qPCR for *HAP43m29/hap43∆* strain, forward  Specific qPCR for *HAP43m29/hap43∆* strain, reverse | TTCACGAAACAATTTAGTGTGAAAG  AGTGTCATCAGAACAAAATCCAC  AAAGAAGAGTGCCTTTGTGAATCT  TAATGTAGACATTGGGTCCATTTG |
| CBO7444 | Specific qPCR for *HAP43/hap43∆* strain, forward | GATTTCACGAAACAATTTAG |
| CBO7445 | Specific qPCR for *HAP43/hap43∆* strain, reverse | CCACAATTGTCAACTGGAGA |
| CBO7446 | Specific qPCR for *HAP43m4/hap43∆* strain, forward | GATTTCACGAAACAATTTGC |
| CBO7447 | Specific qPCR for *HAP43m4/hap43∆* strain, reverse | CCACAATTGTCAACTGGAGC |

**c. Plasmids used in this study.**

| **Plasmid** | **Insert** | **Purpose** | **Reference** |
| --- | --- | --- | --- |
| pSN161 | *PmeI-HAP43 C-terminal ORF sequence-13xMyc-FRT-FLP-SAT1-FRT-HAP43 downstream sequence-PmeI,* *ligated into pRS316 plasmid* | Myc-tagged Hap43 | Noble et al., 2010 |
| pSN219 | *PmeI-SSN3 C-terminal ORF sequence-TAP-FRT-FLP-SAT1-FRT-SSN3 downstream sequence-PmeI, ligated into pRS316 plasmid* | TAP-tagged Ssn3 | Noble et al., 2010 |
| pCB127 | *SacII-5' flank of ADH1-cartTA ORF-SAT1-13xMyc-HAP43 ORF-OP4 promoter-tet operator-3' flank of LEU2-KpnI,* *ligated into pNIM1 plasmid* | tetO-Hap43-Myc | this study |
| pCB193 | *SacII-5' flank of ADH1-cartTA ORF-SAT1-Ubi ORF-3xHA-OP4 promoter-tet operator-3' flank of LEU2-KpnI, ligated into pNIM1 plasmid* | tetO-HA-Ub | this study |
| pCB215 | *SacII-5' flank of ADH1-cartTA ORF-SAT1-13xMyc-HAP43**(S337A,S355A) ORF-OP4 promoter-tet operator-3' flank of LEU2-KpnI, ligated into pNIM1 plasmid* | tetO-Hap43(S337A,S355A)-Myc | this study |
| pCB217 | *SacII-5' flank of ADH1-cartTA ORF-SAT1-13xMyc-HAP43**(S81A) ORF-OP4 promoter-tet operator-3' flank of LEU2-KpnI, ligated into pNIM1 plasmid* | tetO-Hap43(S81A)-Myc | this study |
| pCB218 | *SacII-5' flank of ADH1-cartTA ORF-SAT1-13xMyc-HAP43**(S248A) ORF-OP4 promoter-tet operator-3' flank of LEU2-KpnI, ligated into pNIM1 plasmid* | tetO-Hap43(S248A)-Myc | this study |
| pCB220 | *SacII-5' flank of ADH1-cartTA ORF-SAT1-13xMyc-HAP43**(S498A) ORF-OP4 promoter-tet operator-3' flank of LEU2-KpnI, ligated into pNIM1 plasmid* | tetO-Hap43(S498A)-Myc | this study |
| pCB221 | *SacII-5' flank of ADH1-cartTA ORF-SAT1-13xMyc-HAP43(T610A,T611A) ORF-OP4 promoter-tet operator-3' flank of LEU2-KpnI, ligated into pNIM1 plasmid* | tetO-Hap43(T610A,T611A)-Myc | this study |
| pCB222 | *SacII-5' flank of ADH1-cartTA ORF-SAT1-13xMyc-HAP43**(S620A) ORF-OP4 promoter-tet operator-3' flank of LEU2-KpnI, ligated into pNIM1 plasmid* | tetO-Hap43(S620A)-Myc | this study |
| pCB229 | *SacII-5' flank of ADH1-cartTA ORF-SAT1-13xMyc-HAP43(S87A,S255A) ORF-OP4 promoter-tet operator-3' flank of LEU2-KpnI, ligated into pNIM1 plasmid* | tetO-Hap43(S87A,S255A)-Myc | this study |
| pCB271 | *SacII-5' flank of LEU2-HAP43 terminator-mutant 29 ORF-promoter cassette-ARG4 marker-3' flank of LEU2-KpnI* | *HAP43^mutant 29^* Addback | this study |
| pCB272 | *SacII-5' flank of LEU2-HAP43 terminator-ORF-promoter cassette-ARG4 marker-3' flank of LEU2-KpnI* | *HAP43* Addback | this study |
| pCB284 | *SacII-5' flank of ADH1-cartTA ORF-SAT1-13xMyc-HAP43tr ORF-OP4 promoter-tet operator-3' flank of LEU2-KpnI, ligated into pNIM1 plasmid* | tetO-Hap43tr-Myc | this study |
| pCB298 | *Xma1-HAP43tr-13xMyc-Cla1, ligated into pJK1027 plasmid* | *pACT1-HAP43tr-13xMyc* | this study |
| pCB364 | *Xma1-HAP43m4-13xMyc-Cla1, ligated into pJK1027 plasmid* | *pACT1-HAP43m4-13xMyc* | this study |
| pCB379 | *Xma1-HAP43-13xMyc-Cla1, ligated into pJK1027 plasmid* | *pACT1-HAP43-13xMyc* | this study |
